# Pancreatic RECK inactivation promotes cancer formation, epithelial-mesenchymal transition, and metastasis

**DOI:** 10.1172/JCI161847

**Published:** 2023-09-15

**Authors:** Tomonori Masuda, Akihisa Fukuda, Go Yamakawa, Mayuki Omatsu, Mio Namikawa, Makoto Sono, Yuichi Fukunaga, Munemasa Nagao, Osamu Araki, Takaaki Yoshikawa, Satoshi Ogawa, Kenji Masuo, Norihiro Goto, Yukiko Hiramatsu, Yu Muta, Motoyuki Tsuda, Takahisa Maruno, Yuki Nakanishi, Toshihiko Masui, Etsuro Hatano, Tomoko Matsuzaki, Makoto Noda, Hiroshi Seno

**Affiliations:** 1 Department of Gastroenterology and Hepatology,; 2 Department of Drug Discovery Medicine, Medical Innovation Center,; 3 Division of Hepato-Biliary-Pancreatic Surgery and Transplantation, Department of Surgery, and; 4 Department of Molecular Oncology, Kyoto University Graduate School of Medicine, Kyoto, Japan.

**Keywords:** Gastroenterology, Oncology, Cancer, Mouse models, Tumor suppressors

## Abstract

*RECK* is downregulated in various human cancers; however, how *RECK* inactivation affects carcinogenesis remains unclear. We addressed this issue in a pancreatic ductal adenocarcinoma (PDAC) mouse model and found that pancreatic *Reck* deletion dramatically augmented the spontaneous development of PDAC with a mesenchymal phenotype, which was accompanied by increased liver metastases and decreased survival. Lineage tracing revealed that pancreatic *Reck* deletion induced epithelial-mesenchymal transition (EMT) in PDAC cells, giving rise to inflammatory cancer-associated fibroblast–like cells in mice. Splenic transplantation of *Reck-*null PDAC cells resulted in numerous liver metastases with a mesenchymal phenotype, whereas reexpression of RECK markedly reduced metastases and changed the PDAC tumor phenotype into an epithelial one. Consistently, low RECK expression correlated with low E-cadherin expression, poor differentiation, metastasis, and poor prognosis in human PDAC. RECK reexpression in the PDAC cells was found to downregulate MMP2 and MMP3, with a concomitant increase in E-cadherin and decrease in EMT-promoting transcription factors. An MMP inhibitor recapitulated the effects of RECK on the expression of E-cadherin and EMT-promoting transcription factors and invasive activity. These results establish the authenticity of RECK as a pancreatic tumor suppressor, provide insights into its underlying mechanisms, and support the idea that RECK could be an important therapeutic effector against human PDAC.

## Graphical abstract

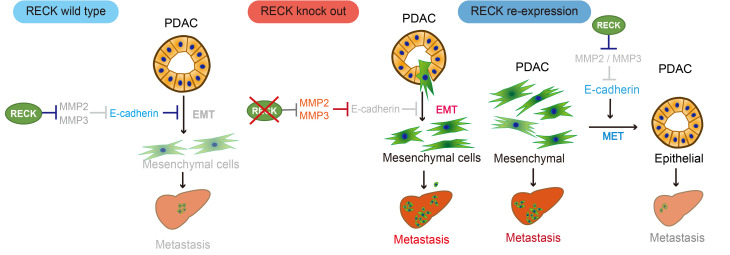

## Introduction

Pancreatic ductal adenocarcinoma (PDAC) is one of the most lethal malignancies. The survival rate for PDAC has remained relatively unchanged for decades, with a 5-year survival rate of approximately 10% (1). This poor prognosis is mainly due to low rates of early diagnosis and the highly invasive and metastatic nature of PDAC. Approximately 50% of patients with PDAC have distant metastases at the time of diagnosis (1).

Many cancer-related deaths are caused by metastasis rather than primary tumors. Metastasis occurs through a sequential, multistep process known as the invasion-metastasis cascade (2, 3). The invasion-metastasis cascade involves several processes, including epithelial-mesenchymal transition (EMT), dissemination, and metastatic colonization (4). However, our understanding of metastasis and its underlying mechanisms remains incomplete. Therefore, it is important to identify molecules that are critical for the processes involved in metastasis and elucidate their action mechanisms.

Reversion-inducing cysteine-rich protein with Kazal motifs (RECK) was originally isolated as a gene that reverts the morphology of transformed cells (5, 6). RECK inhibits multiple members of the MMP family (e.g., MMP2, MMP7, MMP9, and MT1-MMP). MMPs play important roles in tumor invasion and angiogenesis (5, 7–9). In humans, RECK is widely expressed in normal tissues and is reduced in the tissues of many tumor types (10, 11). In some RECK-downregulated cancer cell lines, restoring RECK expression results in reduced invasion and metastasis. *Reck*-deficient mice die on approximately embryonic day 10.5 and show increased gelatinase activity in the tissue, decreased tissue integrity, and abnormalities in vascular and neural development (8, 12, 13).

A previous study indicated that high RECK levels in resected PDAC tissues correlated with better prognoses of patients, implying that RECK plays a tumor-suppressive role in PDAC (14). To date, however, this hypothesis has not been experimentally validated in vivo. In this study, we aimed to clarify this issue using a well-established PDAC mouse model and test the clinical relevance of our findings.

## Results

### Low RECK expression correlates with poor differentiation, metastasis, and worse prognosis in human PDAC.

**Figure 1:**
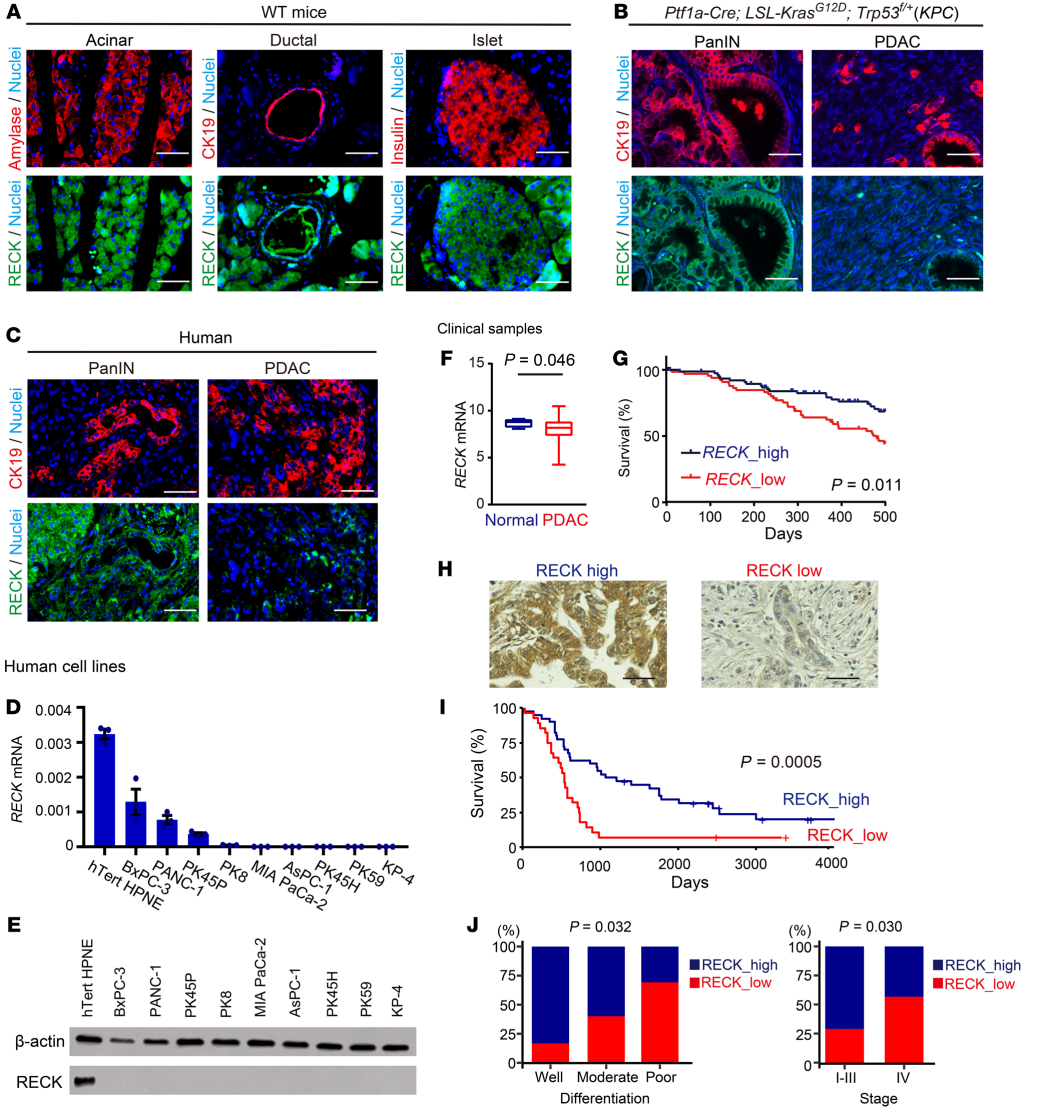
Low *RECK* expression correlates with poor differentiation, metastasis, and worse prognosis in human PDAC. (**A**) Immunofluorescence double staining of pancreatic sections from wild-type mice for RECK (green) and amylase, CK19, or insulin (red) with nuclear counterstaining (Hoechst 33342; blue). Acinar, ductal, and islet cells were examined. Scale bar: 50 μm. (**B**) Immunostaining of pancreatic sections from *KPC* mice for RECK (green) and CK19 (red) with nuclear counterstaining (blue). Scale bar: 50 μm. (**C**) Immunofluorescence double staining of human pancreatic sections containing PanIN or PDAC for RECK (green) and CK 19 (red) with nuclear counterstaining (blue). Scale bar: 50 μm. (**D**) *RECK* mRNA levels in human pancreatic cell lines. RT-qPCR measured RECK mRNA using total RNA, normalized to *Gapdh*. Data are shown as the mean ± SEM of 3 experiments (duplicate). (**E**) RECK protein levels in human pancreatic cell lines. An immunoblot assay detected RECK using total protein from the cell lines, with β-actin as the loading control. (**F**) Comparison of *RECK* mRNA levels between normal human pancreatic tissues and PDACs. TCGA (*n* = 195) data are summarized in box plots. *P* = 0.046 (*t* = –2.8), 2-tailed Welch’s t test. (**G**) Kaplan-Meier survival curves for patients with PDAC with high (*n* = 75) and low *RECK* mRNA expression in tumor tissues (*n* = 76) using TCGA data within a 400-day time frame. *P* = 0.023, log-rank test. (**H**) Immunostaining of human PDAC sections for RECK. Images of sections classified as RECK high and RECK low. Scale bar: 50 μm. (**I**) Kaplan-Meier survival curves for patients with PDAC with high (*n* = 40) and low RECK expression (*n* = 28). *P* = 0.0005, log-rank test. (**J**) Relationship between RECK expression and clinical/histological features of human PDAC samples. Fisher’s exact test revealed significant correlations of RECK expression with histological grade (*P* = 0.032) and stage IV (metastatic disease; *P* = 0.030).

To determine the localization of RECK expression in the pancreas, we first performed immunofluorescence staining for RECK on pancreatic specimens. RECK signals were detected in pancreatic acinar, ductal, and islet cells in adult wild-type mice (Figure 1A). *Ptf1a-Cre; LSL-Kras^G12^; p53^fl/+^* (*KPC*) mice are well established PDAC models. In these mice, RECK signals were clearly detectable in pancreatic intraepithelial neoplasias (PanINs), which are precancerous lesions, but were barely detected in PDAC (Figure 1B). Similarly, in human tissues, the RECK signals were detected in normal pancreatic epithelial cells, including acinar, ductal, and islet cells (data not shown), and in PanINs, but they were hardly detected in PDAC (Figure 1C).

Consistent with these histological observations, RECK mRNA and protein levels were extremely low in several human PDAC cell lines, including BxPC-3, PANC-1, PK45P, PK8, MIA PaCa-2, AsPC-1, PK45H, PK59, and KP-4, as compared with those in the hTERT-immortalized pancreatic epithelial cell line (hTert HPNE) (15) (Figure 1, D and E). Analysis of transcriptomic data from The Cancer Genome Atlas (TCGA) showed that *RECK* mRNA levels were lower in PDAC tissues than in normal pancreatic tissues (Figure 1F).

Consistent with a previous report (14), patients with PDAC with low mRNA levels of *RECK* had significantly lower survival rates than those with high *RECK* mRNA levels (Figure 1G). Similar results were observed when we analyzed tissues from patients with PDAC using immunohistochemistry and compared tumors with low levels of RECK expression to those with high levels of RECK expression (Figure 1, H and I). In addition, low RECK protein expression in these samples significantly correlated with poor differentiation and metastatic property of the tumors (Figure 1J). These data are consistent with the notion that RECK served as a tumor suppressor in human PDAC.

### RECK suppresses PDAC formation in a mouse model.

To assess the role of RECK in normal pancreatic development, we first generated *Ptf1a-Cre; Reck^fl/fl^* (*RC*) mice by crossing *Ptf1a-Cre* mice and *Reck^fl/fl^* mice (Supplemental Figure 1A; supplemental material available online with this article; https://doi.org/10.1172/JCI161847DS1). *RC* mice were born at the Mendelian ratio and did not show any abnormalities in appearance at birth (data not shown). These mice were indistinguishable from control *Ptf1a-Cre* mice in terms of pancreatic weight and morphology (Supplemental Figure 1B). Histologically, RECK was undetected in all acinar cells and approximately half of the ductal cells of *RC* mice (Supplemental Figure 1C). No obvious pancreatic abnormalities were observed, as assessed by H&E staining as well as staining for amylase, cytokeratin 19 (CK19), and insulin (Supplemental Figure 1C). These data indicate that RECK expression by *Ptf1a*-positive (pancreatic epithelial) cells is dispensable for normal pancreatic development.

**Figure 2:**
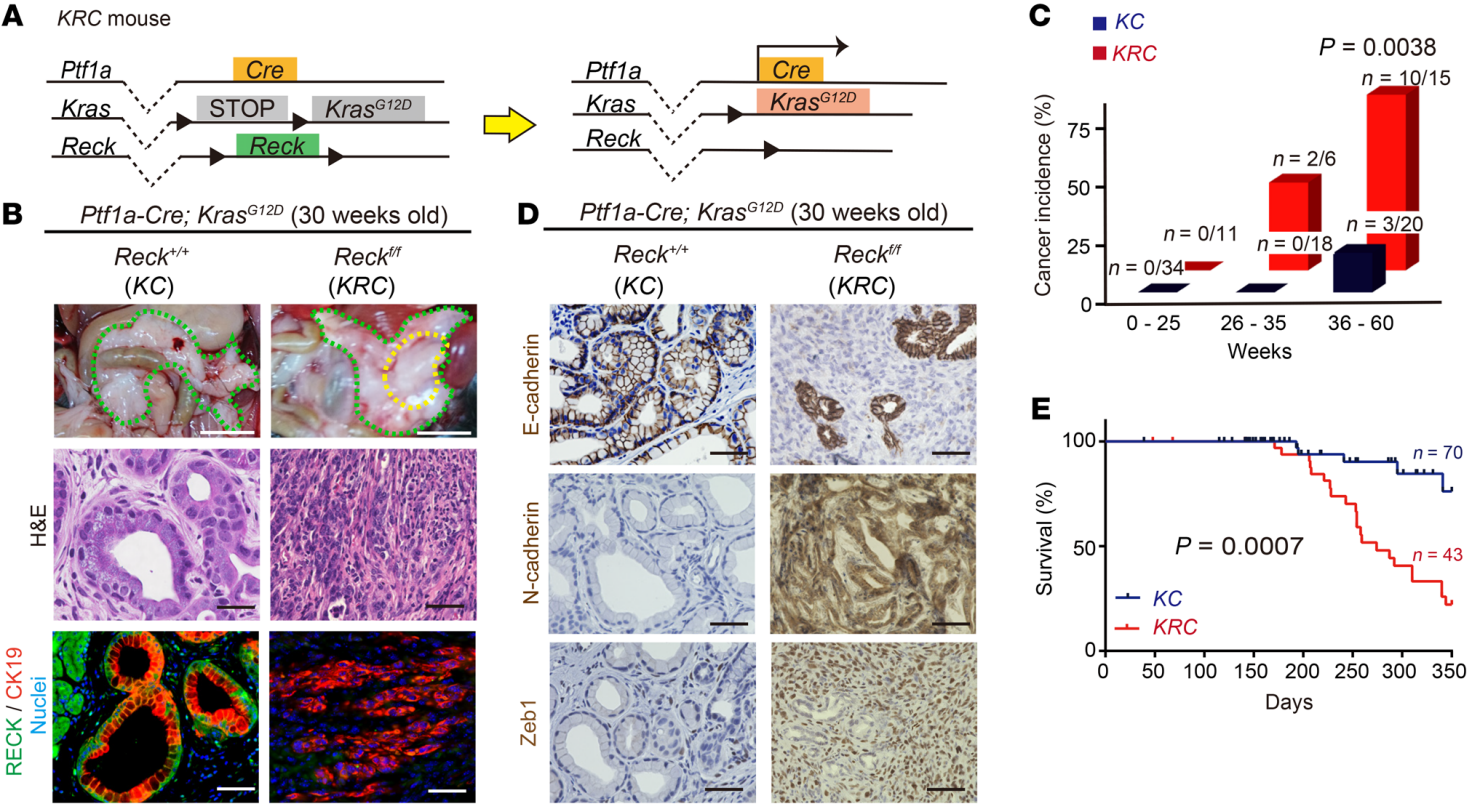
RECK suppresses spontaneous Kras-driven PDAC formation. (**A**) Schematic representation of the Cre-mediated recombination in pancreatic epithelial cells of *KRC* mice. Note that *Ptf1a-Cre* is expressed specifically in the progenitors of pancreatic ductal, exocrine, and endocrine cells (31). (**B**) Macroscopic views of the incised abdomen (scale bar: 10 mm), microscopic images of pancreatic sections after H&E staining, and immunofluorescence double staining for RECK (green) and CK19 (red) followed by nuclear counterstaining (Hoechst 33342; blue). Scale bar: 50 μm. Left column: *Ptf1a-Cre; LSL-Kras^G12D^* (*KC*) mice; right column: *Ptf1a-Cre; LSL-Kras^G12D^; Reck^fl/fl^* (*KRC*) mice. Both groups of mice were at 30 weeks of age. Green dotted lines outline the pancreas, and the yellow dotted line outlines tumor. (**C**) Frequency of PDAC formation in *KC* and *KRC* mice at indicated range of age. *P* = 0.0038 at 36–60 weeks of age, Fisher’s exact test. (**D**) Immunostaining for epithelial and mesenchymal markers in pancreatic tissue sections from *KC* (left) and *KRC* mice (right). E-cadherin, N-cadherin, or Zeb1 were detected followed by hematoxylin counterstaining. Scale bar: 50 μm. (**E**) Kaplan-Meier survival curves for *KC* and *KRC* mice. *n* = 70 (*KC*); *n* = 43 (*KRC*). *P* = 0.0007, log-rank test.

We then moved on to test the effects of pancreatic *Reck* deletion on PDAC formation by generating *Ptf1a-Cre; LSL-Kras^G12D^; Reck^fl/fl^* (*KRC*) mice and comparing them with the control *Ptf1a-Cre; LSL-Kras^G12D^* (*KC*) mice (Figure 2A). At 6 weeks of age, no significant difference was found in the formation of PanIN, as determined by staining with H&E and Alcian blue, between the 2 groups, although the loss of RECK expression in the tissue was evident (Supplemental Figure 2, A and B). Interestingly, the proportions of pRb-positive cells and Ki-67–positive cells among PanIN cells were significantly higher in *KRC* mice compared with those in control *KC* mice, suggesting increased proliferative potential of these cells in the absence of RECK (Supplemental Figure 2C).

Importantly, PDAC developed in some *KRC* mice (*n* = 2 of 6) at 26–35 weeks of age, whereas it did not develop in the control *KC* mice during the same age range (*n* = 0 of 18; Figure 2, B and C). At 36–60 weeks of age, *KRC* mice had significantly higher PDAC formation rates (66.6%, *n* = 10 of 15) than *KC* mice (15.0%, *n* = 3 of 20; Figure 2C). Notably, PDACs in *KRC* mice contained more mesenchymal cells (E-cadherin negative, N-cadherin/Zeb1 positive) than those in *KC* mice (Figure 2D). Furthermore, *KRC* mice exhibited significantly lower survival than *KC* mice (Figure 2E). These data clearly demonstrate that RECK plays an important role in tumor suppression and prevention of PDAC formation and that the lack of RECK somehow results in the formation of PDAC with a mesenchymal phenotype in this mouse model.

### RECK suppresses spontaneous liver metastasis of PDAC in vivo.

**Figure 3:**
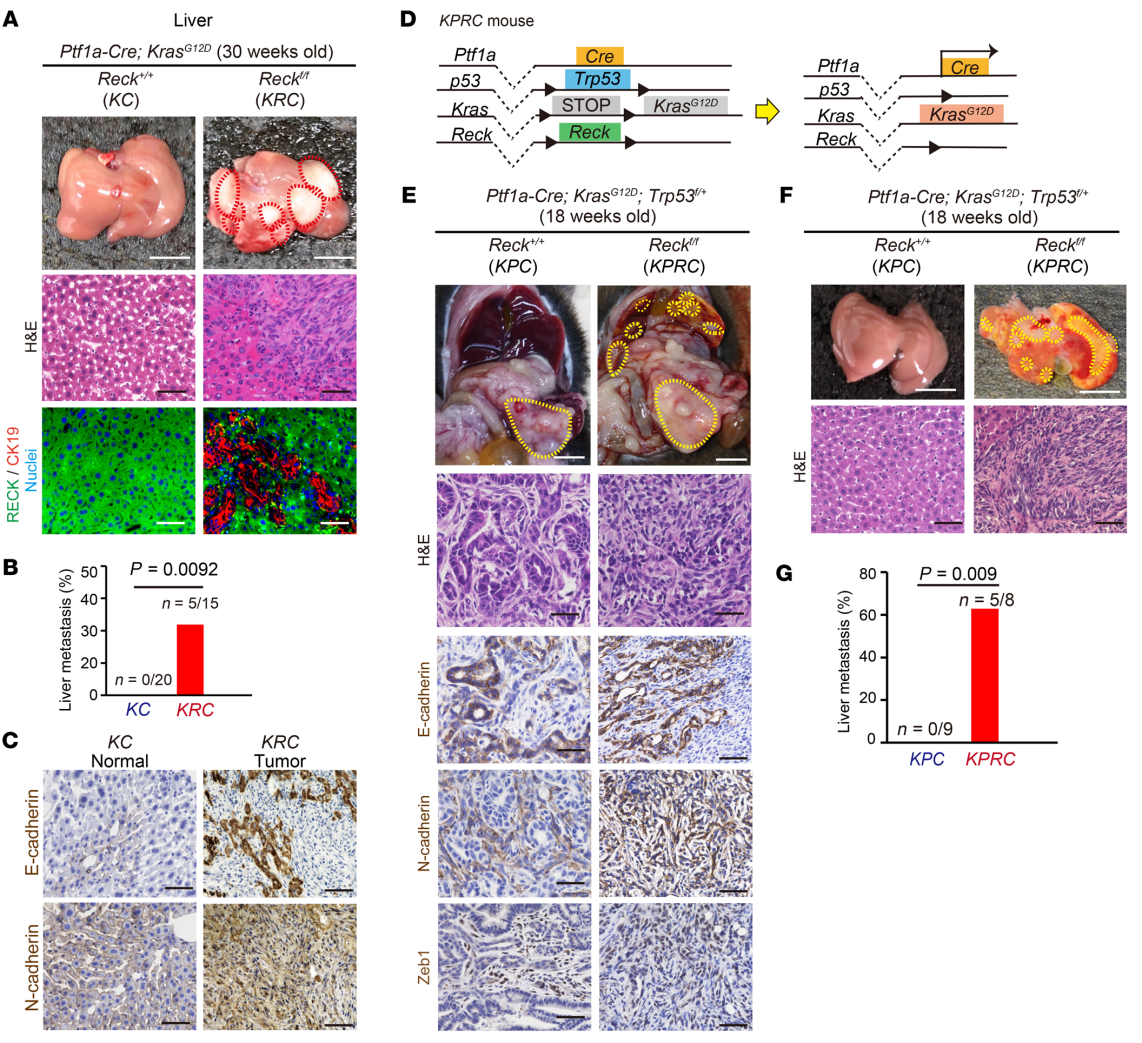
RECK suppresses spontaneous liver metastasis of PDAC. (**A**) Morphology of the excised liver (scale bar: 10 mm) and microscopic images of liver sections after H&E staining or immunofluorescence double staining for RECK (green) and CK 19 (red) followed by nuclear counterstaining (blue). Scale bar: 50 μm. Left column: *KC* mice; right column: *KRC* mice. Red dotted lines outline visible tumors. (**B**) Frequency of tumor metastasis to the liver in *KC* and *KRC* mice. *P* = 0.00925, Fisher’s exact test. (**C**) Immunostaining of epithelial and mesenchymal markers in liver tissues. Liver sections from *KC* (left) and *KRC* (right) mice were immunostained for E-cadherin and N-cadherin, followed by hematoxylin counterstaining. Scale bar: 50 μm. (**D**) Schematic representation of the Cre-mediated recombination resulting in *Kras* activation, *p53* deletion, and *Reck* deletion in pancreatic epithelial cells in *KPRC* mice. Note that this diagram dose not explain the allelic composition. (**E**) Macroscopic views of the incised abdomen (scale bar: 10 mm) and microscopic images of pancreas sections stained with H&E or immunostained for E-cadherin, N-cadherin, or Zeb1, followed by hematoxylin counterstaining. Scale bar: 50 μm. Left column: *KPC* mice; right column: *KPRC* mice. Both groups of mice were at 18 weeks of age. Yellow dotted lines outline visible tumors. (**F**) Morphology of excised liver (scale bar: 10 mm) and images of liver sections after H&E staining (scale bar: 50 μm). Left column: *KPC* mice; right column: *KPRC* mice. Both groups of mice were at 18 weeks of age. (**G**) Frequency of liver metastasis of PDAC in *KPC* and *KPRC* mice. *P* = 0.009, Fisher’s exact test.

To better understand the role of RECK in PDAC progression, we next measured the number of spontaneous liver metastases in *KC* and *KRC* mice. At 30 weeks of age, no liver metastasis was found in *KC* mice with PDAC (*n* = 0 of 20). In contrast, liver metastasis was frequently observed in *KRC* mice with PDAC (33%, *n* = 5 of 15; Figure 3, A and B). Histologically, liver metastases in *KRC* mice also exhibited a mesenchymal phenotype, as characterized by N-cadherin expression (Figure 3C). These data indicate that RECK not only suppresses primary PDAC formation but also suppresses spontaneous liver metastasis of the tumor in this mouse model.

Loss of tumor suppressor p53 is known to accelerate progression and increases the aggressiveness of pancreatic tumors (16). A previous study showed that when combined with a heterozygous p53 background, *Pdx1-Cre; LSL-Kras^G12D^* mice develop PDAC with 100% penetrance (16). To examine the effects of RECK in this system, we generated and compared *Ptf1a-Cre; LSL-Kras^G12D^; Trp53 ^fl/+^; Reck^fl/fl^* (*KPRC*) mice and the control *Ptf1a-Cre; LSL-Kras^G12D^; Trp53 ^fl/+^* (*KPC*) mice (Figure 3D). At 16–24 weeks of age, PDAC was observed in all mice of both groups. Histological features of PDAC developed in *KPRC* mice indicated that these tumors are of the mesenchymal phenotype. As compared with the tumors in *KPC* mice, the tumors in *KPRC* mice contained fewer E-cadherin–positive cells and more N-cadherin–positive cells and Zeb1-positive cells (Figure 3E). Notably, we frequently found liver metastases in *KPRC* mice (62.5%, *n* = 5 of 8), whereas no liver metastases were found in the control *KPC* mice (*n* = 0 of 9; Figure 3, F and G). In this system, however, no significant difference in survival rates between *KPC* and *KPRC* mice was found (Supplemental Figure 3), probably due to the devastatingly rapid growth of original tumors in this system that would mask the effects of metastasis on the survival of animals. Nevertheless, these results clearly indicate that the loss of RECK expression is required for spontaneous liver metastases of PDAC even in the context of heterozygous *p53* deletion.

### RECK suppresses EMT in pancreatic epithelial cells in mice.

**Figure 4:**
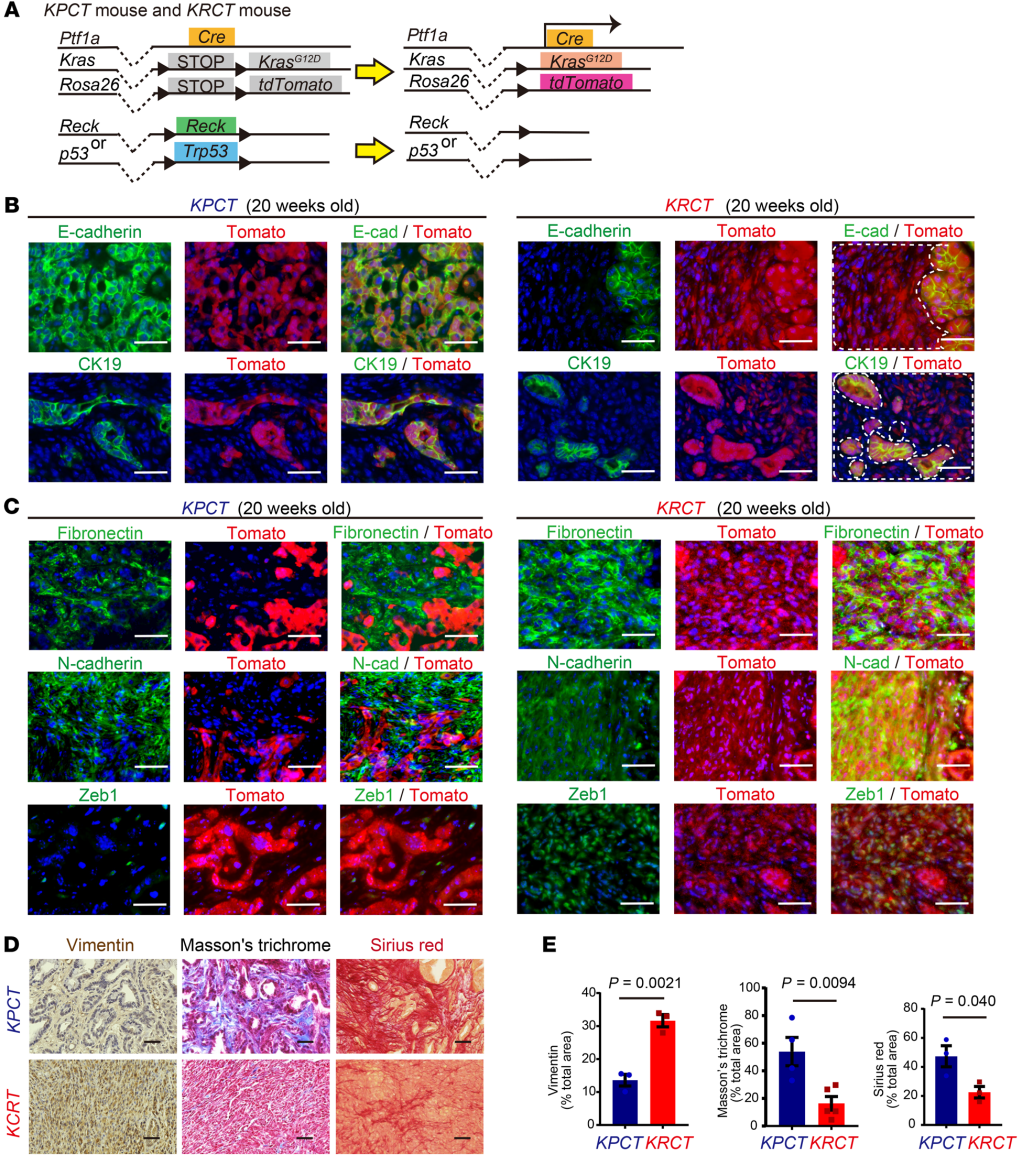
Epithelial-mesenchymal transition occurs in *Reck*-null PDAC that developed in *KRCT* mice. (**A**) Schematic representation of *Ptf1a-Cre*–mediated recombination in pancreatic cells in the lineage-tracing experiments using *Ptf1a-Cre; LSL-Kras^G12D^; Trp53^fl/+^*; *LSL-Rosa^td-tomato^* (*KPCT*) and *Ptf1a-Cre; LSL-Kras^G12D^; Reck^fl/fl^*; *LSL-Rosa^td-tomato^* (*KRCT*) mice. Pancreatic epithelial cells are tagged by the red fluorescent protein tdTomato. This diagram dose not explain allelic composition. (**B**) Immunofluorescence double staining for lineage marker Tomato (red; Ptf1a-expressing cells) and epithelial markers (green), E-cadherin or CK19, with nuclear counterstaining (blue) in PDAC sections from *KPCT* (left 3 columns) and *KRCT* mice (right 3 columns). Images from the same field excited for green (epithelial cells) and red fluorescence (pancreatic epithelium-derived cells) are shown separately (left 2 panels) and overlaid (right panel) on images of blue fluorescence (indicating nuclei). White dotted lines mark the border between red cells (nonepithelial cells derived from pancreatic epithelial cells) and yellow cells (epithelial cells derived from pancreatic epithelial cells). (**C**) Immunofluorescence double staining for mesenchymal markers (green), fibronectin, N-cadherin, and Zeb1, with lineage marker Tomato (red) and nuclear counterstaining (blue) in PDAC sections from *KPCT* (left 3 columns) and *KRCT* mice (right 3 columns). Red pancreatic epithelium-derived cells coexpressing green mesenchymal marker (fibronectin, N-cadherin, or Zeb1) are yellow in the overlay panels (right columns), and such cells are abundant in PDAC that developed in *KRCT* mice. (**D**) Immunostaining for vimentin and staining with Masson’s trichrome or Sirius red. Top row: *KPCT* mice; bottom row: *KRCT* mice. (**E**) Morphometric quantification of vimentin-positive area (pan-CAF), area stained blue with Masson’s trichrome (collagen fibers) and Sirius red–positive area (collagen fibers). Data are shown as the mean ± SEM of the data obtained from 3 sections each from 3 mice. Vimentin-positive area, *P* = 0.0021; Masson’s trichrome, *P* = 0.0094; Sirius red, *P* = 0.040; 2-tailed Student’s *t* test.

Mesenchymal cells found in PDACs developed in *Reck* mutant mice can be either cancer-associated fibroblasts (CAFs) or tumor cells that have undergone EMT. To gain insight into the nature and origin of these cells, we performed lineage tracing experiments in *Ptf1a-Cre; LSL-Kras^G12D^; Reck^fl/fl^*; *LSL-Rosa^td-tomato^* (*KRCT*), *Ptf1a-Cre; LSL-Kras^G12D^; Trp53 ^fl/+^*; *Reck^fl/fl^*; *LSL-Rosa^td-tomato^* (*KPRCT*), and *Ptf1a-Cre; LSL-Kras^G12D^; Trp53 ^fl/+^*; *LSL-Rosa^td-tomato^* (*KPCT*) mice (Figure 4A and Supplemental Figure 4A). In these animals, Cre recombination induces Tomato expression in all cells originated from *Ptf1a*-expressing cells (i.e., pancreatic epithelial cells). In *KRCT* and *KPCT* mice, almost all Tomato-positive cells in the premalignant PanIN lesions were positive for epithelial markers and negative for mesenchymal markers (Supplemental Figure 5 and data not shown). In PDACs, however, we found a striking difference in the properties of Tomato-positive cells between these lines. In the control *Reck* wild-type, *p53*-null PDACs (Figure 4, B and C, left), almost all Tomato-positive (red) cells were positive for epithelial markers (E-cadherin and CK19; Figure 4B) and negative for mesenchymal markers (N-cadherin and Zeb1; Figure 4C). In contrast, in *Reck*-null PDACs, Tomato was expressed in both epithelial cells (Figure 4B, yellow cells, and Supplemental Figure 4B, yellow cells) and mesenchymal cells (Figure 4C, yellow cells, and Supplemental Figure 4C, yellow cells). Fibronectin, which is produced by mesenchymal stromal cells in PDAC (17), was more abundant and more closely associated with Tomato-positive cells in *R*eck-null PDAC than in *Reck* wild-type, *p53*-null PDAC (Figure 4C and Supplemental Figure 4C, yellow cells).

These data indicate that the mesenchymal cells found in PDACs developed in *Reck* mutant mice originated from pancreatic epithelial cells.

### Pancreatic RECK deletion gives rise to iCAF-like cells derived from pancreatic epithelial cells via EMT in PDAC lesions.

The mesenchymal cells in *Reck*-null PDAC showed fibroblastic morphology and were positive for vimentin (Supplemental Figure 6A and Supplemental Figure 7A). Despite the desmoplastic nature of *Reck*-null PDACs, Masson’s trichrome and Sirius red staining suggested that collagen fibers were less abundant in *Reck*-null PDACs compared with those in *Reck* wild-type and *p53*-null PDACs (Figure 4, D and E, and Supplemental Figure 4D).

It is known that both tumor-promoting iCAFs (αSMA-low) and the tumor-suppressive myofibroblast-associated cancer-associated fibroblast (myCAFs) (αSMA high) are present in PDACs (18). We therefore asked whether the pancreatic-epithelium-derived mesenchymal cells found in *Reck*-null PDACs share any characteristics with these CAFs by performing double immunofluorescence staining for the *Ptf1a*-lineage marker (Tomato; red) and for a series of CAF markers (green; Supplemental Figure 6, A and B, and Supplemental Figure 7, A and B), including vimentin (panCAF), αSMA (myCAF), and IL-6 (iCAF). Given that PDGFRb is another marker for panCAF, we also performed double immunofluorescence staining for PDGFRb and vimentin. Almost all vimentin-positive cells were also positive for PDGFRb in PDACs of *KRCT*, *KPRCT*, and *KPCT* mice (Supplemental Figure 6, A and B, and Supplemental Figure 7, A and B), supporting the notion that vimentin is a panCAF marker. In the PDACs of *KPCT* mice, vimentin, αSMA, and IL-6 did not colocalize with Tomato, indicating that no CAF-like cells are derived from pancreatic epithelial cells in PDAC developed in *KPCT* mice. In contrast, many red cells in *Reck*-null PDACs were positive for vimentin and IL-6 but negative for αSMA (Supplemental Figure 6A and Supplemental Figure 7A), indicating that these cells share a similar expression pattern of marker proteins with iCAF. These results suggest that pancreatic *Reck* deletion gives rise to iCAF-like cells derived from pancreatic epithelial cells via EMT.

### The effects of RECK to suppress metastatic potential and mesenchymal phenotype of PDAC cells are cell-autonomous and reversible.

**Figure 5:**
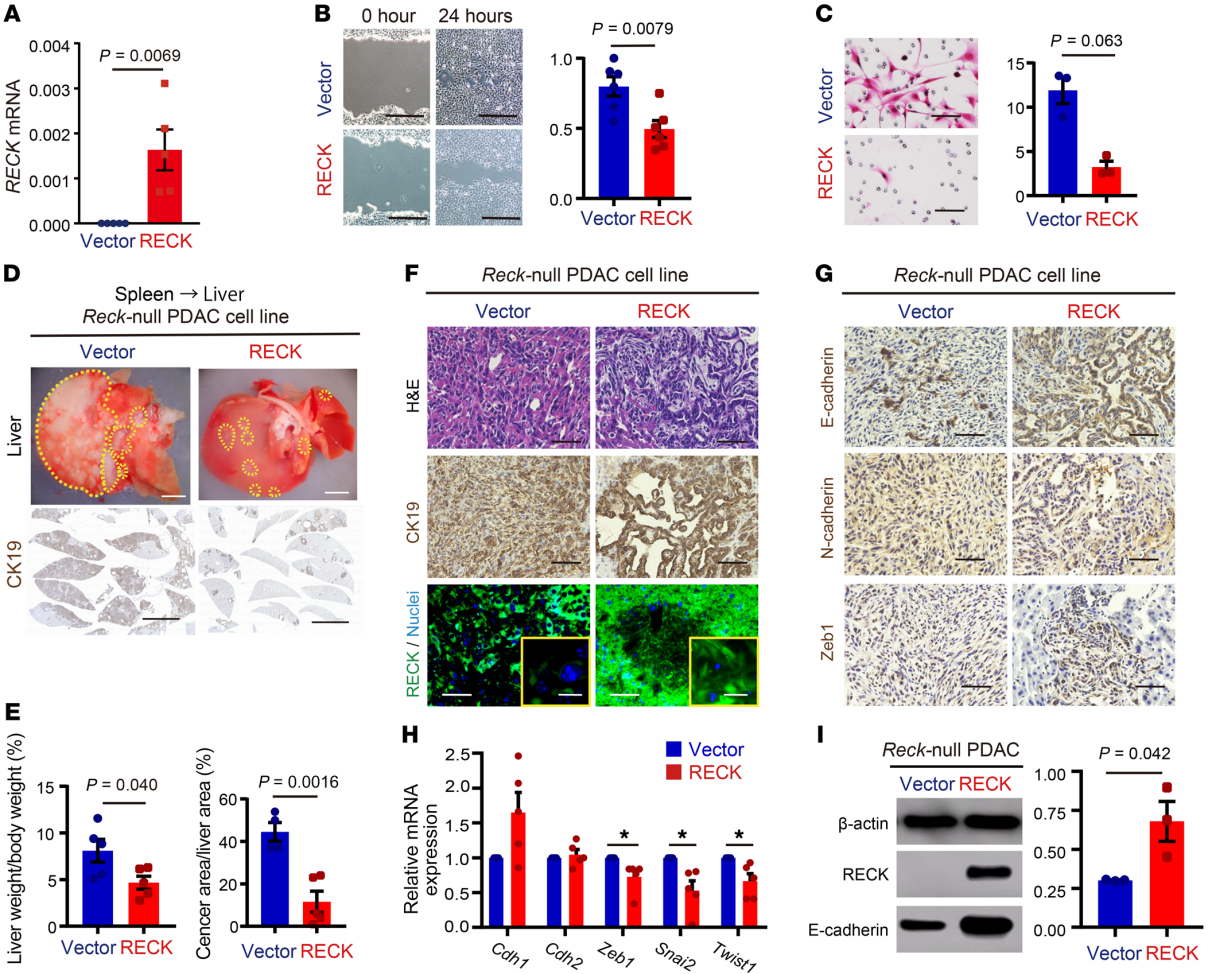
Effects of RECK reexpression on *Reck*-null PDAC cell lines. (**A**) RECK reexpression in *Reck*-null PDAC cell lines. *Reck*-null PDAC cells from *KRC* mice were transfected with control or RECK expression vector. Exogenous *RECK* expression was confirmed by RT-qPCR. Data are shown as the mean ± SEM. *P* = 0.0069, 2-tailed Student’s *t* test. (**B**) Effects of RECK on PDAC cell migration. Wound healing assay using *Reck*-null PDAC cell lines transfected with control or RECK expression vector. Left: Images of the scratched zone and recovered wound 24 hours after scratch (scale bar: 500 μm). Right: Data are shown as the mean ± SEM of the migrated area relative to control. *P* = 0.0079, 2-tailed Student’s *t* test. (**C**) Effects of RECK expression on PDAC cell invasion. Matrigel invasion assay using *Reck*-null PDAC cells (*n* = 3) transfected with control or RECK expression vector. Left: Images of migrated cells (scale bar: 50 μm). Right: Data are shown as the mean ± SEM of the invasion ratio. *P* = 0.0063, 2-tailed Student’s *t* test. (**D**–**G**) Effects of RECK on PDAC cell metastasis. (**D**) Morphology of resected liver (scale bar: 5 mm) and CK19 immunostaining (scale bar: 50 μm). (**E**) Quantitative assessments of liver metastasis. Ratio of liver weight to body weight and CK19-positive area. Data are shown as the mean ± SEM. *P* = 0.040 and *P* = 0.0016, respectively; 2-tailed Student’s *t* test. (**F**) Histology of liver lesions. H&E staining, or immunostaining for CK19 or RECK with nuclear counterstaining (blue) (scale bar: 50 μm). (**G**) Immunostaining for E-cadherin, N-cadherin, or Zeb1 (scale bar: 50 μm). (**H**) Effects of RECK on epithelial and mesenchymal marker gene expression in PDAC cells. RT-qPCR analysis of mRNA levels. Data are shown as the mean ± SEM. *P* = 0.022 (*Zeb1*)*, P* = 0.008 (*Snai2*)*,* *P* = 0.015 (*Twist1*); 2-tailed Student’s *t* test, *P < 0.05. (**I**) Effects of RECK on E-cadherin protein by immunoblot assay. Data are shown as the mean ± SEM of the densitometry measurements of the immunoblot band. *P* = 0.042, 2-tailed Student’s *t* test.

To examine the cell-autonomous effects of RECK on PDAC cells, we established cell lines from PDACs developed in *KRC* mice (*RECK-*null PDAC cell lines) and reexpressed RECK in these cells by retroviral gene transfer; control cell lines infected with empty retroviral vectors were also prepared (Figure 5A). Migration and invasion activities, but not proliferation rate (data not shown), were significantly reduced upon RECK reexpression (Figure 5, B and C).

When transplanted into the spleens of wild-type mice, the vector-transfected cells gave rise to numerous macroscopic liver metastases (Figure 5D), whereas RECK-reexpressed cells gave rise to markedly fewer liver metastases (Figure 5, D and E). Histological examination revealed that metastatic lesions formed after transplantation of vector-transfected cells contained numerous mesenchymal cells (E-cadherin positive, N-cadherin/Zeb1 negative) that invaded the surrounding liver parenchyma (Figure 5, F and G, left). In contrast, *RECK*-transfected cells that had metastasized to the liver exhibited epithelial morphology with strong CK19/E-cadherin immunoreactivity and were localized in confined areas (Figure 5, F and G, right). These data with clonal cell lines indicate that it is the RECK in PDAC cells, rather than the RECK in stromal cells, that affects the metastatic potential of tumors. Of note, multiple EMT-promoting transcription factor genes, such as *Zeb1*, *Snai2*, and *Twist1*, were downregulated in *RECK*-transfected cells (Figure 5H), suggesting that RECK suppresses EMT of these cells. Interestingly, RECK upregulated E-cadherin protein (Figure 5I) but not its mRNA (Cdh1 in Figure 5H), raising the possibility that RECK regulates E-cadherin expression posttranscriptionally. These data also indicate that the effects of RECK on PDAC cells is reversible.

### RECK downregulates genes related to EMT, MYC targets, and E2F targets in PDAC cells.

**Figure 6:**
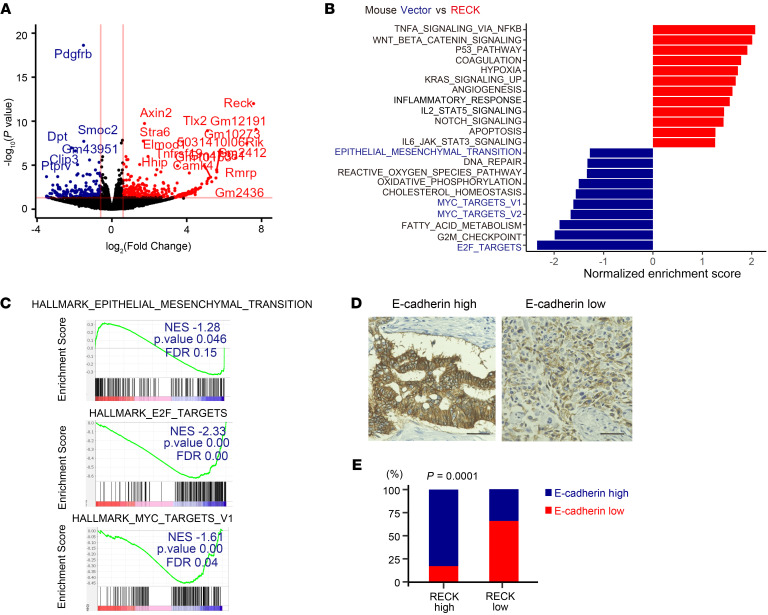
Genes related to EMT, MYC targets, and E2F targets are downregulated after reexpression of RECK in *Reck-*null PDAC cells. (**A**) Volcano plot of RNA-Seq data. Genes downregulated in RECK-transfected PDAC cells (log [fold change] < –0.6 and *P* < 0.05) are shown in blue, and the upregulated genes (log [fold change] > 0.6 and *P* < 0.05) are shown in red. (**B**) Hallmark gene sets significantly upregulated (red) or downregulated (blue) in RECK-transfected PDAC cells, as assessed by GSEA. Gene set names presented in blue on the y-axis indicate gene sets that correspond to the experimental phenotype under study. (**C**) Examples of Hallmark gene sets downregulated in RECK-transfected PDAC cells compared with those in the control vector–transfected (*Reck*-null) cells. (**D**) Immunohistochemistry detects E-cadherin in human PDAC specimens. Microscopic images of human PDAC sections classified as E-cadherin high and E-cadherin low are shown. Scale bar: 50 μm. (**E**) Relationship between RECK expression and E-cadherin expression in human PDAC specimens. Sections of PDAC tissues from 69 patients were subjected to immunohistochemical staining for RECK (see Figure 1H) and E-cadherin (see **D**). Cases were classified into 4 groups as shown in Figure 6E and evaluated by Fisher’s exact test. Note the significant positive correlation between RECK expression and E-cadherin expression (*P* = 0.0001).

To gain more insights into the molecular mechanisms underlying the suppression of EMT and metastasis by RECK, we compared the transcriptomes of 3 PDAC cell lines transfected with either the control vector or RECK expression vector. We identified 93 differentially expressed genes (Supplemental Table 1), many of which were related to the cell membrane and extracellular matrix (Figure 6A). Gene set enrichment analysis (GSEA) revealed that RECK negatively regulated the expression of gene sets termed E2F_TARGETS, MYC_TARGETS_V1, and EPITHELIAL_MESENCHYMAL_TRANSITION (Figure 6, B and C), which is consistent with our observation that RECK suppressed PDAC formation, EMT, and metastasis in mice. Because these data (Figure 6, A–C) were obtained using RNA from cultured PDAC cells, our findings further support the idea that RECK affects EMT in a cell-autonomous manner.

To start testing the clinical relevance of our findings, we stained a set of human PDAC tissues (*n* = 69) with anti-RECK and anti–E-cadherin antibodies. The results indeed indicated a significant correlation between RECK expression and E-cadherin expression in human PDAC (Figure 6, D and E).

### RECK downregulates MMP2/MMP3 and an MMP inhibitor recapitulates the effects of RECK on cell invasion and the levels of E-cadherin protein and EMT-promoting transcription factor mRNAs in PDAC cells.

**Figure 7:**
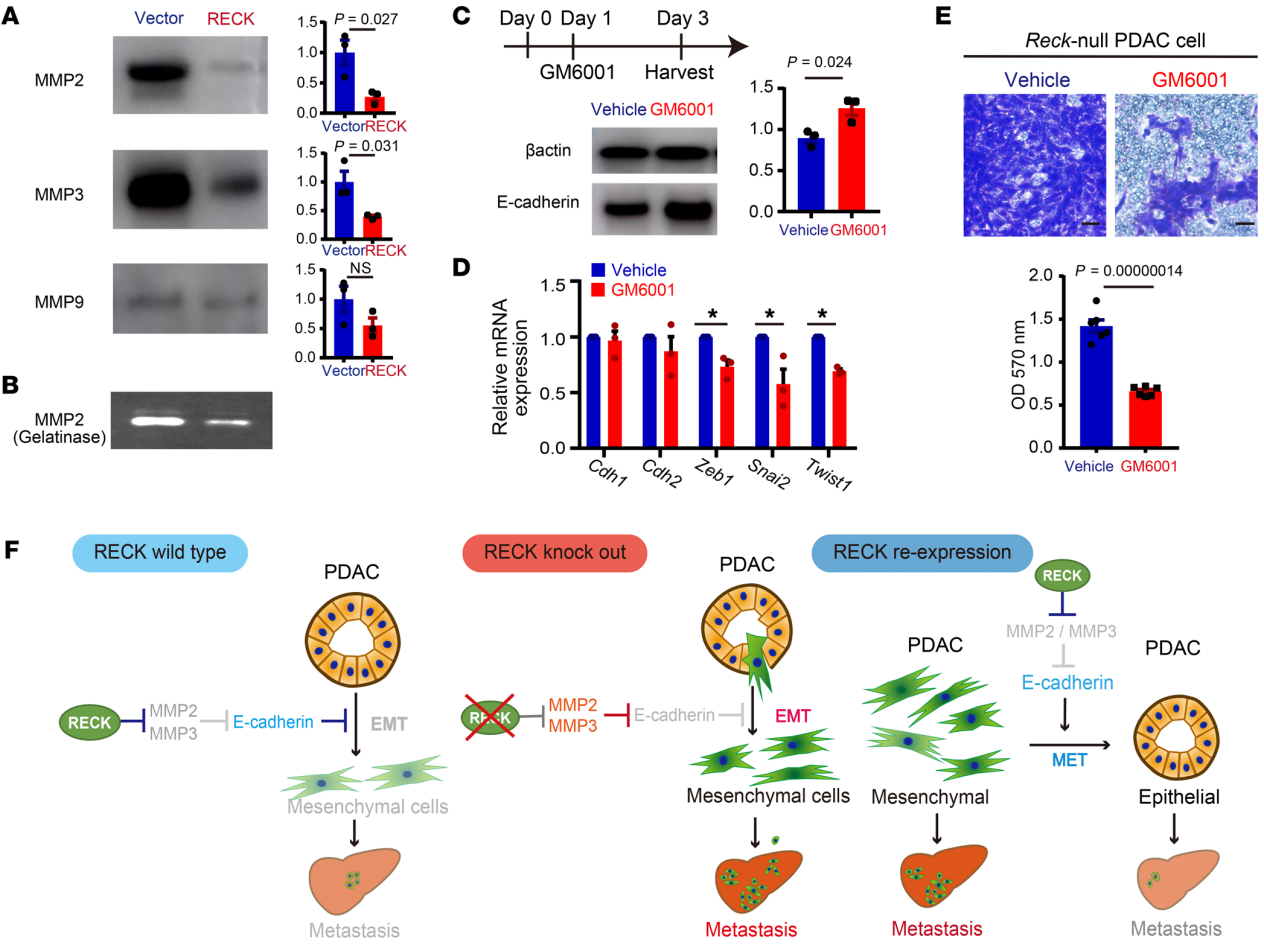
Involvement of MMPs in RECK-induced upregulation of E-cadherin and suppression of EMT in PDAC cells. (**A**) Detection of MMP2, MMP3, and MMP9 in culture supernatant of *Reck*-null PDAC cells transfected with control or RECK expression vector by immunoblot assay. Note that we adjusted the sample volume based on cell number counted at the time of harvest for direct comparison. Data are shown as the mean ± SEM of the densitometry measurements of the immunoblot band. *P* = 0.027 (MMP2), *P* = 0.031 (MMP3), *P* = 0.155 (MMP9); 2-tailed Student’s *t* test. (**B**) Detection of 72 kDa gelatinase (MMP2) in culture supernatant by gelatin zymography. Culture supernatants as used in **A** were subjected to gelatin zymography. (**C**) Effects of an MMP inhibitor (GM6001) on E-cadherin expression in *Reck*-null PDAC cells. Cells treated as shown in the top diagram were subjected to immunoblot assay using antibodies against β-actin (loading control, top) and E-cadherin (bottom). Data are shown as the mean ± SEM of the densitometry measurements of the immunoblot band. *P* = 0.024; 2-tailed Student’s *t* test. (**D**) Effects of GM6001 on EMT marker genes. Total RNA from the cells as used in **C** was subjected to RT-qPCR to estimate the levels of indicated mRNAs. Data are shown as the mean ± SEM. *P* = 0.01 (*Zeb1*); *P* = 0.033 (*Snai2*); *P* = 0.00009 (*Twist1*), 2-tailed Student’s *t* test. (**E**) Effects of GM6001 on PDAC cell invasion. Matrigel invasion assay was performed using *Reck*-null PDAC cell lines (*n* = 6) treated with either DMSO or GM6001. Top: Images of cells that migrated through the Matrigel insert membrane after staining (blue). Scale bar: 50 μm. Bottom: Data are shown as the mean ± SEM of the optical density (OD) at 570 nm. *P* = 0.00000014; 2-tailed Student’s *t* test. (**F**) Models consistent with our findings.

RECK is known to regulate several MMPs, including MMP2 and MMP9 (5). In our efforts to assess the contribution of MMPs to the EMT found in *Reck*-null PDACs, we tried to detect several MMPs in culture supernatant of *Reck*-null and RECK-reexpressed PDAC cells by immunoblot assay and found that the levels of MMP2 and MMP3 were decreased after RECK reexpression, while the level of MMP9 remained comparable to that of the control (Figure 7A); the decrease of MMP2 in RECK-reexpressed PDAC cells could also be detected by gelatin zymography (Figure 7B). The observed downregulation of MMP3 was of particular interest, because E-cadherin is known to be a substrate of this protease (19–22). When *Reck*-null PDAC cells were treated with a broad-spectrum MMP inhibitor, GM6001, E-cadherin was upregulated at the protein level (Figure 7C) but not at the mRNA level (Cdh1 in Figure 7D), and EMT-promoting transcription factors, Zeb1, Snai2, and Twist1, were downregulated at the mRNA level (Figure 7D). Furthermore, GM6001 significantly reduced the invasion activity of *Reck*-null PDAC cells (Figure 7E). The effects of GM6001 in *Reck*-null PDAC cells were reminiscent of that of RECK reexpression (Figure 5, C, H, and I), suggesting that RECK suppresses EMT and increases E-cadherin protein expression by regulating MMP(s). Since E-cadherin is known to regulate the expression of EMT-related genes (23), these data are consistent with the model that E-cadherin is a primary target of MMPs whose degradation leads to EMT and that RECK prevents MMP-catalyzed degradation of E-cadherin, thereby suppressing EMT and consequent metastasis of PDAC cells (Figure 7F).

## Discussion

*RECK* downregulation occurs in many cancer types and is often correlated with a poor prognosis (14, 24–29). In some *RECK*-downregulated cancer cell lines, restoration of *RECK* expression results in reduced invasion (5, 8). These observations suggest that *RECK* is a tumor suppressor gene; however, the actual role of RECK in carcinogenesis in vivo remains largely unknown. In the present study, we combined pancreatic *RECK* deletion and genetic lineage tracing in the well-established PDAC mouse model and made 3 major observations. First, pancreatic *RECK* inactivation markedly increased both spontaneous development of PDAC and its metastasis to the liver, providing the first direct evidence to our knowledge that *RECK* is a bona fide tumor suppressor. Second, pancreatic *RECK* inactivation confers a mesenchymal phenotype to PDAC. Third, experiments using PDAC-derived cell lines revealed that RECK suppresses the invasive and metastatic abilities of PDAC cells in a cell-autonomous manner. In addition, experiments using *Reck*-null PDAC cell lines in vitro suggested that RECK suppresses EMT by upregulating E-cadherin, at least in part, through downregulation of MMP2 and MMP3. Histologic and bioinformatic studies on clinical samples revealed that low *RECK* expression correlated with low E-cadherin expression, poor differentiation, metastasis, and worse prognosis in human PDAC, supporting the clinical relevance of our findings in mice and cell lines.

Since RECK is known to be downregulated by various oncogenes, including mutant *RAS* (5), we initially hypothesized that RECK should be already downregulated in *Kras^G12D^*-induced premalignant lesions, PanINs, as well as in invasive PDAC. We found, however, that RECK was readily detectable in PanINs, but not in PDAC, in both human and mouse specimens (Figure 1C and Supplemental Figure 2A). These findings indicate that RAS mutations are not sufficient for effective RECK silencing and that RAS mutations and RECK silencing are independent steps toward PDAC formation. The exact mechanism by which *RECK* silencing is induced during progression from PanIN to PDAC is therefore an important issue remains to be elucidated.

A previous study showed that RECK does not affect the growth of sarcoma cells in xenograft experiments (5). This suggests that RECK suppresses cancer metastasis and recurrence rather than tumor growth. In this study, however, we found that pancreatic *RECK* inactivation resulted in a marked increase in the incidence of *Kras^G12D^*-induced PDAC formation. We found that *RECK* expression had little effect on the incidence of PanIN formation (Supplemental Figure 2, A and B), which indicates that RECK suppresses PDAC formation by blocking the progression from PanIN to PDAC. Mechanistically, our RNA-Seq data showed that the expression of MYC targets and E2F targets was upregulated in *Reck*-null PDAC cells compared with that in the RECK-reconstituted PDAC cells (Figure 6C). Interestingly, proportions of pRb-positive cells and Ki-67–positive cells were significantly higher in PanINs developed in *KCR* mice compared with those in control *KC* mice (Supplemental Figure 2C), raising the possibility that *RECK* deletion results in increased proliferative potential of PanIN cells through E2F activation, thereby increasing the probability of tumor progression.

Application of lineage tracing to the study of pancreatic cancer progression was pioneered by Rhim, Stanger, et al. (30), who found that EMT and dissemination precede pancreatic tumor formation and that inflammation plays important roles in these processes. In the present study, however, mesenchymal cells of pancreatic epithelial origin were undetected in PanIN lesions. A major difference between our study and the previous report (30) was the use of different Cre-driver mice, *Pdx1-Cre* and *Ptf1a-Cre*. Since *Pdx1* expression precedes *Ptf1a* expression and shows wider distribution than that of *Ptf1a* (31, 32), this could be a cause of the discrepancy between the results of two studies. Although our data presented here provide little evidence supporting the relevance of RECK to tissue inflammation besides the emergence of iCAF-like cells in *Reck*-null PDACs, our preliminary data indicate that the *RECK* gene is negatively regulated by NF-κB signaling (our unpublished observations), which may implicate RECK downregulation in the long-sought signal that triggers EMT of pancreatic epithelial cells in response to tissue inflammation.

Our results with *KPC* mice (Figure 3, D–G) suggest that RECK plays a role independent of p53 during PDAC progression. RNA-Seq analysis showed that *PDGFRb* is one of the most upregulated genes in *Reck*-null PDAC cells. Of note, PDGFRb is known to play a critical role in PDAC metastasis induced by a gain-of-function mutant of *p53* (*p53^R172H^*) in the *KC* mouse system (33). In the *KPC* mice we used in this study (Figure 5, E–G), Cre recombination induces heterozygous deletion of p53 gene, and complete loss of its tumor suppressor activity relies on a spontaneous second-hit mutation on the wild-type allele (a great majority of these mutations are expected to be loss of heterozygosity), which is sufficient to induce PDAC at 100% penetrance but insufficient to promote metastasis. It is therefore tempting to speculate that *Reck* inactivation in *KC* mice brings about a similar outcome in effect as does *p53^H172R^* to activate PDGFR signaling, thereby increasing tumor incidence as well as metastasis. This hypothesis needs to be tested in future studies.

Pancreatic *Reck* deletion was found to result in the emergence of αSMA-low/IL-6–high mesenchymal (vimentin-positive) CAF-like cells via EMT of PDAC cells (Supplemental Figures 6 and 7), which share a similar expression pattern of marker proteins with a tumor-promoting type of CAF, termed inflammatory CAF (iCAF) (18, 34). The nature and the clinical importance of iCAF-like cells that emerge in the absence of RECK warrant further investigation.

Despite the desmoplastic morphology of *Reck*-null PDAC tissues, decreased abundance of collagen fibers after histological staining was found (Figure 4D and Supplemental Figure 5D). Since myCAFs are known to produce collagen fibers (35), the increase in iCAF-like cells in *Reck*-null PDAC tissues may account for the phenomenon. Our RNA-Seq data, however, indicated no upregulation of fibrillar collagen genes by RECK reexpression (data not shown). Although this may suggest the irreversible nature of the transformation from PDAC cells to the iCAF-like cells, collagen abundance may also be altered by posttranscriptional mechanisms. For instance, the observed downregulation of MMP2 by RECK in *Reck*-null PDAC cells (Figure 7, A and B) may suggest deregulated collagenolysis in *Reck*-null PDAC tissues. Previous observation that global *Reck* knockout results in fragile embryos with reduced type I collagen immunoreactivity (8) supports this model.

Restoring RECK expression in *Reck-*deficient PDAC cells markedly increased E-cadherin expression and suppressed mesenchymal morphology, mesenchymal marker expression, and the invasive and metastatic potential of PDAC cells. These results indicate that the EMT triggered by *Reck* deletion in PDAC is a cell-autonomous and reversible process. Given that RECK expression is downregulated in the majority of human PDAC, RECK could be an important therapeutic effector against human PDAC and compounds that stimulates RECK expression (36, 37) could be a novel therapeutic drug for this dismal disease.

How does RECK sustain E-cadherin expression? Since the ability of MMP3 to cleave E-cadherin has been reported (19–22), our data so far support the model that RECK downregulates MMP3, thereby upregulating E-cadherin and suppressing EMT and the mesenchymal phenotype (Figure 7F). However, the actual involvement of MMP3 (as well as MMP2) and E-cadherin in the action of RECK in the context of PDAC has to be rigorously tested in future studies. In addition, the questions as to whether MMP2 cleaves E-cadherin, whether these MMPs digest any other substrate(s) critical for regulating EMT, how RECK downregulates MMP2 and MMP3, whether any other MMP family members are involved in this phenomenon, and what is the relationship between the effects of RECK on EMT and on E2F and MYC remain to be clarified.

In conclusion, we found that pancreatic *RECK* inactivation markedly promotes both spontaneous development of PDAC and its liver metastasis in the PDAC mouse models, establishing RECK as a bona fide tumor suppressor. In addition, pancreatic *RECK* inactivation induces EMT in PDAC cells. Mechanistically, our data suggest that RECK suppresses EMT by downregulating MMP2 and MMP3 and, thereby, increasing E-cadherin expression in PDAC cells. Consistently, low RECK expression correlated with low E-cadherin expression, poor differentiation, metastasis, and poor prognosis in human PDAC. Therefore, RECK should be considered as an important therapeutic effector against human PDAC.

## Methods

### Animal experiments.

All animals were maintained in a specific pathogen–free facility. All surgical manipulations were performed under isoflurane anesthesia, and every effort was made to minimize suffering. Experimental animals were generated by crossing *Ptf1a-Cre* mice (a gift from Yoshiya Kawaguchi, Kyoto University) (31) with mice harboring *LSL-Kras^G12D^* (a gift from David Tuveson, Cold Spring Harbor Laboratory (Cold Spring Harbor, New York, USA) (38), *Reck*^fl/fl^ (39), *p5*3^fl/+^ (purchased from The Jackson Laboratory, JAX strain 008462), or LSL-*Rosa^td-tomato^* (The Jackson Laboratory, JAX strain 007909). The mice were crossed in a mixed background with no selection for a specific sex.

### Primary mouse PDAC cells in culture.

PDAC tissues resected from *KRC* mice were minced with scissors. After digestion with 2.5 mg/mL collagenase D (Roche) at 37°C for 15 minutes with agitation, the tissues fragments were further dissociated with a gentleMACS dissociator (Miltenyi Biotec). The cells were passed through a 100 μm cell strainer, pelleted by centrifugation at 100*g* for 5 minutes, suspended, and plated on a dish in DMEM containing 10% FBS and 50 U/mL penicillin-streptomycin. Cultures were maintained at 37°C in a humidified atmosphere of 5% CO_2_ /95% air.

### Retroviral infection.

The control retroviral vector (LXSB) and the vector expressing RECK have been described previously (40). After viral infection in the presence of 8 μg/mL polybrene, stable transfectants were selected for 10 days with 8 μg/mL blasticidin-S, starting from 24 hours after infection. After selection, the levels of RECK in the pooled population of transfectants were assessed by immunoblot assay as previously described. To prepare culture supernatants for immunoblot assay and gelatin zymography, cells were plated (1 × 10^6^ in 60 mm dish) and incubated for 48 hours in 5 mL DMEM containing 10% FBS and then in 1 mL serum-free DMEM for 24 hours. The same volume of culture supernatants from vector-transfected cells and RECK-transfected cells were used for these assays. Final cell numbers at the time of sample harvest were similar between 2 groups of cells (difference ≤20%).

### Human PDAC cell lines.

The BxPC-3 (CRL-1687), PANC-1 (CRL-1469), MIA Paca-2 (CRL-1420), and AsPC-1 (CRL-1682) cell lines were obtained from ATCC. Additionally, the PK45P (RCB2141), PK45H (RCB1973), PK8 (RCB2700), PK59 (RCB1901), and KP-4 (RCB1005) cell lines were purchased from RIKEN Cell Bank (RCB), and maintained under the same conditions as PDAC cell lines. The control hTERT-immortalized pancreatic epithelial cell line (hTert HPNE) (15) was obtained from ATCC.

### Clinical samples.

Gene expression data and clinical information for patients with PDAC were obtained from publicly available TCGA database. The mRNA expression levels of the target gene were extracted from the TCGA data set. To divide patients with PDAC into high (*n* = 75) and low (*n* = 76) expression groups, we used the median of *RECK* mRNA expression level as the cutoff value. Kaplan-Meier survival analysis was performed for the survival curves for high and low *RECK* mRNA expression groups. The survival time for each patient was measured from the date of diagnosis to the date of death or the last follow-up. Survival curves were plotted using a 400-day time frame.

Surgically resected specimens of human pancreatic cancer tissues were obtained from patients who had been admitted to Kyoto University Hospital. Immunohistochemistry staining for RECK protein was performed. Patients were categorized into 2 groups, high (*n* = 40) and low (*n* = 28), based on their RECK protein expression, as determine by immunohistochemistry. Kaplan-Meier survival analysis was performed for the survival curves for high and low RECK protein expression groups. The log-rank test was used to evaluate the statistical significance of the differences in survival outcomes between the high and low RECK protein expression groups. Additionally, χ^2^ or Fisher’s exact test was performed to analyze the association between protein expression levels and clinicopathological parameters.

### Histology.

For immunohistochemistry, tissues were perfused with and fixed in 4% paraformaldehyde in PBS, dehydrated in 70% ethanol, embedded in paraffin, and sectioned at 5 μm thickness. Standard protocols were used for H&E, Masson’s trichrome, and Sirius red staining. For immunohistochemistry, antigen unmasking was performed by incubating the sections in citric acid buffer (pH 6.0) or EDTA buffer (pH 8.0) for 15 minutes at 98°C in a microwave oven. Blocking was performed by incubating the sections with a blocking solution (Dako). The primary antibodies used in this study were as follows: mouse anti-RECK (5) (1:100), rabbit anti-CK19 (1:100; ab52625; Abcam), rabbit anti-Rb(phosphor S780) (1:100; ab47763; ab47763), rabbit anti-Ki67 (1:100; ab1667; Abcam), rabbit anti–E-cadherin (1:100; 3195; Cell Signaling Technology), rabbit anti–N-cadherin (1:100; 13116; Cell Signaling Technology), rabbit anti-zeb1 (1:1,000; MPA027524; Sigma-Aldrich), rabbit anti-fibronectin (1:100; ab2413; Abcam), rabbit anti-αSMA (1:100; ab5694; Abcam), rabbit anti-IL-6 (1:100; ab6672; Abcam), rabbit anti–NF-κB p65(1:100; 4764s; Cell Signaling Technology), rabbit anti-PDGFRβ (1:100; ab23914; Abcam), mouse anti-vimentin (1:100; sc-6260 AC; Santa Cruz Biotechnology), and goat anti-RFP (1:100; 200-101-379; Rockland). Secondary antibodies used were as follows: goat anti-rabbit (1:200; BA-1000; Vector Laboratories), goat anti-guinea pig (1:200; BA-7000; Vector Laboratories), horse anti-mouse (1:200; BA-2000; Vector Laboratories), CF647 goat anti-mouse (1:200, 20262; Biotium), CF488A donkey anti-mouse (1:200; 20014; Biotium), donkey anti-goat, Alexa Fluor 555 (1:200, A21432; Thermo Fisher Scientific), goat anti-mouse, Alexa Fluor 488 (1:200; A11001; Thermo Fisher Scientific), goat anti-rabbit, Alexa Fluor 555 (1:200; A21428; Thermo Fisher Scientific), donkey anti-rabbit, Alexa Fluor 488 (1:200; A21206; Thermo Fisher Scientific), and donkey anti-mouse, Alexa Fluor 488 (1:200; A32766; Thermo Fisher Scientific). Sections were incubated with primary antibodies for 2 hours at room temperature or overnight at 4°C, washed, and then incubated with secondary antibodies for 1 hour at room temperature. Immune complexes were visualized using the ABC Kit (Vector Laboratories) and DAB Kit (Dako), followed by counterstaining with hematoxylin. For immunofluorescence staining, sections were counterstained with Hoechst 33342 (Thermo Fisher Scientific). PanIN was visualized by Alcian blue staining of the sections, and its size on the micrographs was quantified using ImageJ software 1.0 (NIH). Five complete pancreatic sections were analyzed.

### Immunoblot assay.

Cells were lysed with RIPA buffer (20 mM Tris-HCl, 37 mM NaCl, 2 mM EDTA, 1% Triton X-100, 0.1% SDS, 0.5% sodium deoxycholate, and 10% glycerol). Lysates were mixed with SDS sample buffer (Nacalai Tesque, 09499-14) and heated to 95°C for 5 minutes. Culture supernatant was collected as described in retroviral infection procedures. The supernatant was mixed with SDS sample buffer (Nacalai Tesque, 09499-14) and heated to 95°C for 5 minutes. Both cell lysates and culture supernatants were subjected to SDS–polyacrylamide gel electrophoresis using 5%–15% gradient gels. Proteins were transferred onto polyvinylidene difluoride membranes (Bio-Rad, 1704156) using the Trans-Blot Turbo Transfer System (Bio-Rad) on the “Mixed MW” setting. The membranes were then incubated with blocking buffer (Blocking One; Nacalai Tesque, 03953-95) for 1 hour at room temperature (25°C) and then with the indicated primary antibodies overnight at 4°C. Next, the membranes were washed in 0.05% Tris-buffered saline and 0.1% Tween 20 buffer and incubated with HRP-conjugated secondary antibodies (1:2,000, Cell Signaling Technology, 7074 for anti-rabbit IgG, 7076 for anti-mouse IgG) for 1 hour. Bands were detected with the Super Signal West Pico PLUS Chemiluminescent Substrate (Thermo Fisher Scientific, 34577) and recorded with an Amersham Imager 600 (GE Life Sciences). The following primary antibodies were used for immunoblotting: mouse anti-β-actin (1:10,000; MA1140; Thermo Fisher Scientific), mouse anti-RECK (5) (1:3,000), rabbit anti–E-cadherin (1:1,000; 3195; Cell Signaling Technology), rabbit anti-MMP2 (1:1,000; 40994; Cell Signaling Technology), rabbit anti-MMP3 (1:1,000; 14351; Cell Signaling Technology), rabbit anti-MMP9 (1:1,000; ab38898; Abcam).

### Gelatin zymography.

Gelatinases (MMP2 and MMP9) in culture supernatant were detected by gelatin zymography using AK47 Zymography Kit (Cosmo Bio). Ten μL cell culture supernatants was mixed with nonreducing SDS-sample buffer and loaded onto a 10% SDS-polyacrylamide gel containing 0.1% gelatin. After electrophoresis, the gel was washed with 2.5% Triton X-100 to remove SDS and then incubated in reaction buffer at 37°C for 24 hours. Finally, the gel was stained with Coomassie blue and destained until the clear bands of gelatinolysis became visible.

### Wound healing assay.

Cells were seeded (5 × 10^5^/well) in 6-well tissue culture plates. Once at the confluence, the cell layer was scratched with a 200 μL pipette tip, and images were taken using a phase-contrast microscope (scratched area). After incubation for 24 hours, the same part of the dish was photographed to quantify the recovered wound area. Data are presented as the migration area ratio (recovered wound area divided by scratched area). Experiments were performed in triplicate, and 3 wells were used per group.

### Metastasis assay.

For experimental metastasis assays, C57BL/6 mice (Charles River) were anesthetized by continuous isoflurane gas inhalation and incised under the left rib to expose the spleen. Then, 2 × 10^5^ viable cells suspended in 0.1 mL PBS were injected into the spleen. Fourteen days after inoculation, the livers of the mice were inspected for metastatic nodules, and histological examination was performed.

### RNA isolation and RT-qPCR.

RNA was isolated using the RNeasy Kit (QIAGEN). Complementary DNA was synthesized using the ReverTra Ace qPCR RT Kit (Toyobo). PCR was performed with a SYBR Green–based (Roche) gene expression assay using the LightCycler 96 System (Roche). *Gapdh* was used as the normalizing control. Primers were designed using the Massachusetts General Hospital PrimerBank (https://pga.mgh.harvard.edu/primerbank/). All reactions were performed in duplicate.

### Gene expression analysis.

Three cell lines were established independently from PDACs developed in 3 *KRC* mice. The cell lines were infected with either a vacant LXSB virus (control) or an LXSB virus carrying *RECK* cDNA, followed by the selection of stable transfectants, as described above. Total RNA was isolated from 6 PDAC cell populations (3 vector transfected and 3 RECK transfected). RNA-Seq and library preparation were performed by Macrogen. Raw RNA-Seq data were processed using the STAR (version 2.7.4a) (41) and RSEM (version 1.3.2) (42) softwares. Differential expression analysis was performed using the edgeR (version 3.36.0) (43–45) software package (R package). Normalization was performed using the trimmed mean of M values. A likelihood ratio test was performed between the RECK and control groups, and the genes with *q* < 0.30 were selected as differentially expressed genes. Pathway analysis was performed by GSEA (version 4.2.2) (46, 47) using preranked lists against data sets in the MSigDB database (https://www.gsea-msigdb.org/gsea/msigdb). To identify RECK-associated genes in human PDAC samples, RNA-Seq data from 183 human PDAC samples were downloaded from the TCGA database. RECK-high and RECK-low samples were defined as samples that showed RECK expression levels higher or lower than the mean value, respectively. The expression profiles of RECK-high and RECK-low samples were subjected GSEA using hallmark gene sets.

### MMP inhibition assay.

*Reck*-null PDAC cells (1.0 × 10^5^ /well) were seeded onto 6-well plates and allowed to attach overnight. Medium was then replaced with fresh medium containing GM6001 (30 μM; BIO 5192; R&D Systems) or DMSO, and the cells were incubated for 48 hours. After incubation, the cells were used for immunoblot assay, RT-qPCR, or invasion assay.

### Invasion assay.

For invasion assay of RECK-reexpressed PDAC cells, cells (2 × 10^5^) were suspended in serum-free DMEM and placed in the top compartment of a BioCoat Matrigel insert or control insert. The bottom compartment was filled with DMEM containing 10% FBS, and, after incubation for 22 hours at 37°C in a CO_2_ incubator, the number of penetrating cells was counted. The invasion ratio (the number of cells that had migrated through the Matrigel insert divided by the number of cells that had migrated through the control insert) was calculated. For invasion assay of *Reck*-null PDAC cells treated with MMP inhibitor, cells (2 × 10^5^) were suspended in serum-free DMEM containing 30 μM GM6001 or DMSO (vehicle) and placed in the top insert of CytoSelect 24-well Cell Migration and Invasion Assay (8 μm), Colorimetric, Combo Kit (CBA-100; Cell Biolabs). The lower compartment was filled with DMEM containing 10% FBS. After incubation for 36 hours at 37°C in a CO_2_ incubator, invaded cells were stained with cell stain solution (part 11002) and quantified by measuring the optical density at 570 nm after extraction with extraction solution (part 11003).

### Statistics.

Statistical tests used for evaluating particular data are mentioned in figure legends and include 2-tailed Welch’s *t* test, log-rank test, Fisher’s exact test, χ^2^ test, and 2-tailed Student’s *t* test. Most quantitative data are presented as mean ± SEM. All statistical analyses were performed and Kaplan-Meier survival curves drawn, using the R software (version 4.0.5). Statistical significance was set at *P* < 0.05, and 2-tailed Student’s *t* tests were employed for comparisons.

### Study approval.

All animal experiments were approved by the Animal Research Committee, Graduate School of Medicine, Kyoto University, and performed in accordance with Japanese government regulations. Surgically resected specimens of human pancreatic cancer tissues were obtained from patients who had been admitted to Kyoto University Hospital. The study protocol (no. G1200-1, R2904) was approved by the Ethics Committee of Kyoto University Hospital.

### Data availability.

The RNA-Seq data used in this study have been deposited in the DNA Data Bank of Japan Sequence Read Archive (accession DRA013528). The processed RNA-Seq data have been deposited in the Genomic Expression Archive (accession E-GEAD-478). Microscopy data reported in this paper will be shared by AF upon request. Values for all data points in graphs are reported in the Supporting Data Values file.

## Author contributions

T Masuda and AF conceived and designed the study. T Masui and EH provided clinical samples. T Masuda, GY, T Matsuzaki, MO, M Namikawa, MS, YF, M Nagao, OA, TY, SO, KM, NG, YH, YM, MT, T Maruno, and YN conducted the experiments. T Masuda wrote the manuscript, and AF, M Noda, and HS revised it.

## Supplementary Material

Supplemental data

Supporting data values

## Version 1 09/15/2023

Electronic publication
