# Supplementary material for: Pancreatic RECK inactivation promotes cancer formation, epithelial-mesenchymal transition, and metastasis
Source: J Clin Invest. 2023 Sep 15;133(18):e161847. doi: 10.1172/JCI161847 (PMC10503799; doi:10.1172/JCI161847)
Supplement: Supplemental data [file jci-133-161847-s244.pdf]

Supplemental Table 1. List of differentially expressed gens (DEGs).

Gene	logFC	logCPM	LR	p value	FDR
Rpl27-ps3	11.747	3.320	431.731	6.820E-96	9.620E-92
Rpl10-ps1	8.809	0.446	83.115	7.740E-20	5.460E-16
Pdgfrb	-1.520	5.155	82.308	1.160E-19	5.470E-16
Reck	7.574	-0.703	51.190	8.390E-13	2.950E-09
Axin2	1.746	2.371	39.734	2.910E-10	8.200E-07
Dbn1	0.565	5.674	32.910	9.650E-09	2.270E-05
Smoc2	-1.503	4.746	31.911	1.610E-08	3.030E-05
Ier3	0.508	6.137	31.785	1.720E-08	3.030E-05
Stra6	1.685	3.090	31.152	2.390E-08	3.740E-05
Dpt	-2.157	2.552	27.178	1.860E-07	2.615E-04
Gm43951	-1.956	2.672	26.139	3.180E-07	4.070E-04
Zc3h11a	0.753	6.132	24.463	7.580E-07	0.0008897
Vcam1	-0.495	6.737	23.726	1.110E-06	0.0012045
Elmod1	1.931	2.089	23.474	1.270E-06	0.0012743
Spns2	0.858	3.835	22.217	2.440E-06	0.0022155
Serpinb9b	0.795	5.049	22.155	2.520E-06	0.0022155
Krt20	-1.191	3.526	21.320	3.890E-06	0.0032217
Cd34	-0.535	7.434	20.778	5.160E-06	0.0038368
Arhgap27	0.580	4.625	20.772	5.170E-06	0.0038368
Atg9b	0.641	5.321	20.530	5.870E-06	0.0041371
Cxcl12	-0.683	7.263	19.981	7.820E-06	0.0052491
Foxf1	-1.840	2.730	19.634	9.380E-06	0.0060082
Cadps	1.488	2.067	19.480	1.020E-05	0.0062292
Gsta4	-0.546	6.015	18.950	1.340E-05	0.0078786
Lepr	0.771	3.978	18.699	1.530E-05	0.0086304
Htra1	0.369	6.302	17.673	2.620E-05	0.0142171
Hhip	2.408	0.782	17.419	3.000E-05	0.0156497
Avil	0.567	6.632	17.141	0.0000347	0.0169791
Lgals2	-0.872	4.509	17.128	0.0000349	0.0169791
Mcpt1	2.619	-0.847	16.550	0.0000474	0.0222599
Slc4a4	0.524	5.734	15.936	0.0000655	0.0297837
Dusp1	0.533	4.955	15.779	0.0000712	0.0313576
mt-Co3	-0.654	8.204	15.646	0.0000764	0.0326246
Adh7	-0.765	4.035	15.541	0.0000808	0.0334739
Sema7a	0.408	5.779	15.149	0.0000993	0.0400064
Gm50241	-1.334	2.987	15.050	0.0001047	0.0403451
Csrp2	-0.488	5.369	15.028	0.0001059	0.0403451
Zc3h11a	-0.649	6.156	14.827	0.0001179	0.0437129
Stc1	2.339	2.947	14.705	0.0001257	0.0445254
Tgfbi	-1.198	2.981	14.685	0.0001270	0.0445254
Epas1	-1.160	3.250	14.649	0.0001295	0.0445254
Tafa1	-1.806	1.676	14.341	0.0001525	0.0502595
Rps18-ps3	-1.537	0.623	14.300	0.0001559	0.0502595
Fst	0.867	3.141	14.287	0.0001569	0.0502595
Ces1g	-1.869	1.974	14.103	0.0001731	0.0542026
Galnt18	-0.613	4.291	14.040	0.0001790	0.0548425
Scn5a	1.048	3.068	13.979	0.0001849	0.0554410
Shisa2	1.000	2.875	13.775	0.0002060	0.0604928
Rbp1	-0.519	4.703	13.241	0.0002739	0.0787867

Crisp1	1.499	1.222	12.934	0.0003227	0.0897400
Procr	0.430	5.646	12.922	0.0003247	0.0897400
Pcdh19	0.479	6.401	12.826	0.0003418	0.0920045
Csf1	-0.358	6.927	12.803	0.0003460	0.0920045
H2bc8	1.401	0.046	12.383	0.0004331	0.1130515
Srpx2	1.865	1.567	12.324	0.0004471	0.1145610
Ctsc	0.627	6.487	12.251	0.0004650	0.1170188
Pdpn	0.932	3.402	12.043	0.0005198	0.1270005
Gm10132	0.629	3.364	12.033	0.0005226	0.1270005
Rgs2	0.777	3.331	11.991	0.0005347	0.1277354
Acat2	-0.328	7.316	11.947	0.0005474	0.1285915
Atoh8	-0.511	4.428	11.889	0.0005646	0.1288290
Aldh3a1	-0.810	5.227	11.882	0.0005667	0.1288290
Gm11518	-0.966	5.216	11.728	0.0006156	0.1343219
Gm8995	-2.221	0.244	11.721	0.0006179	0.1343219
Flnc	0.564	6.554	11.698	0.0006258	0.1343219
Igfbp4	0.279	10.766	11.672	0.0006346	0.1343219
Tecpr1	0.367	7.297	11.660	0.0006385	0.1343219
Wnt5a	0.567	4.044	11.555	0.0006757	0.1400498
H3c11	2.778	-1.721	11.376	0.0007440	0.1519615
4933427D14Rik	0.508	4.523	11.265	0.0007898	0.1590141
Lgals4	-0.460	6.164	10.810	0.0010095	0.1990855
1700012B09Rik	0.585	4.287	10.796	0.0010170	0.1990855
Nostrin	-1.380	0.704	10.730	0.0010539	0.2034720
Car5b	0.365	5.024	10.693	0.0010756	0.2048541
Kcnh1	1.142	2.115	10.569	0.0011501	0.2143795
Mmp13	1.644	0.812	10.559	0.0011560	0.2143795
Prickle1	0.405	4.588	10.502	0.0011925	0.2182818
Rpl30-ps9	-4.128	0.931	10.454	0.0012239	0.2211485
Zfand2a	0.344	6.283	10.419	0.0012473	0.2225303
Adnp	0.291	6.264	10.372	0.0012796	0.2254327
Ltbp2	-0.339	6.482	10.156	0.0014380	0.2481581
Zfp697	0.449	4.055	10.149	0.0014438	0.2481581
Gm20431	-3.615	0.662	10.018	0.0015506	0.2615501
Apln	-0.392	5.281	9.988	0.0015757	0.2615501
Shank2	1.179	0.988	9.965	0.0015956	0.2615501
Cldn3	1.169	2.645	9.954	0.0016049	0.2615501
Gm3776	-0.851	3.340	9.943	0.0016145	0.2615501
Gzmd	1.851	0.135	9.846	0.0017025	0.2700967
CT010467.1	0.335	9.924	9.840	0.0017078	0.2700967
Marchf11	1.623	1.278	9.822	0.0017248	0.2700967
St8sia6	1.407	2.478	9.718	0.0018248	0.2826220
Wnt7a	1.460	3.129	9.665	0.0018779	0.2876864
Myo5b	0.790	5.000	9.590	0.0019568	0.2965484

FC, fold change; CPM, counts per million; LR, likelihood ratio; p value was calculated by edgeR package of R software; FDR, false discovery rate.

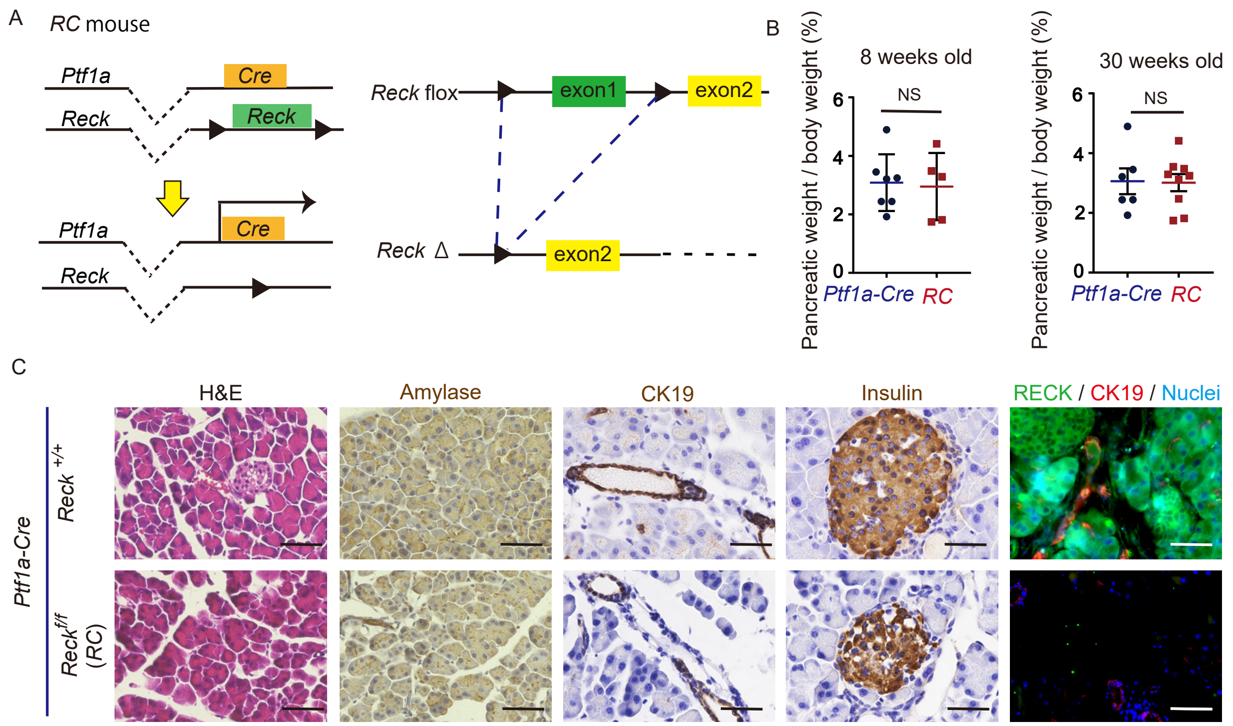

Supplemental Figure 1. RECK is dispensable for pancreatic development. (A) Left part: Schematic representation of the Cre-mediated recombination in pancreatic epithelial cells in *Ptf1a-Cre; Reck$^{f/f}$ (RC)* mice. Right part: In the *Reck^{f}* allele that we used, exon 1 is flanked by two loxP sites (represented as arrowheads) so that Cre recombination between two loxP sites results in the deletion of exon 1 and hence a lack of intact RECK protein. (B) Ratio of pancreatic weight to body weight of *Ptf1a-Cre* (control) and *Ptf1a-Cre; Reck$^{f/f}$* (*RC*) mice. Left panel: *Ptf1a-Cre* (n = 6) and *RC* mice (n = 5) at 8 weeks of age. Right panel: *Ptf1a-Cre* (n = 6) and *RC* mice (n = 9) at 30 weeks of age. Blue and red horizontal lines: mean. Error bars: SEM. NS: non-significant (p > 0.05, Student’ s t-test). (C) Representative images of pancreatic tissues stained with H&E or immunostained for amylase, CK19 (red), insulin, or RECK (green), and nuclei (Heochest33342, blue) (scale: 50 μm). Upper panels: *Ptf1a-Cre* mice. Lower panels: *RC* mice.

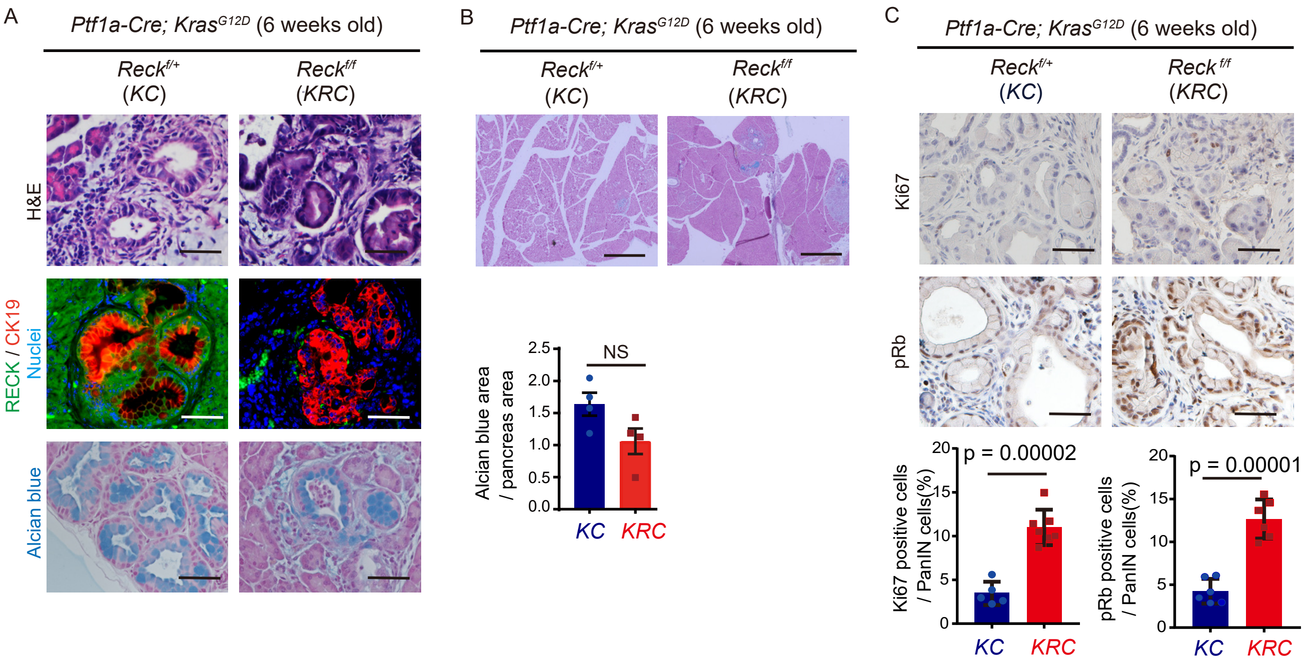

Supplemental Figure 2. Effects of pancreatic *Reck* deletion on PanIN formation. (A) Representative images of pancreatic tissues stained with H&E, immunostained for RECK (green), CK19 (red), and nuclei (Heochest33342; blue), or stained with alcian blue (scale: 50 μm). Left panel: control *Ptf1a-Cre; LSL-Kras^{G12D}; Reck$^{f/+}$ (KC)* mice. Right panels: *Ptf1a-Cre; LSL-Kras^{G12D}; Reck$^{f/f}$ (KRC)* mice. (B) Quantification of PanIN area. Low-magnification images (scale: 500 μm) of pancreatic tissues from *KC* or *KCR* mice stained with alcian blue. Bottom graph: the ratio of alcian-blue-positive area (PanIN) to total area was determined using the ImageJ software1.0. Bar represents mean ± SEM of data obtained from three sections each from three *KC* mice and four *KCR* mice. (C) Left part: Representative images of pancreatic tissues immunostained for Ki-67 or pRb (scale: 50 μm). Left panels: control *KC* mice. Right panels: *KRC* mice. Bottom graph: the ratio of Ki-67 positive cells or pRb positive cells (PanIN). Bar represents mean ± SEM of data obtained from three sections each from six *KC* mice and seven *KRC* mice.

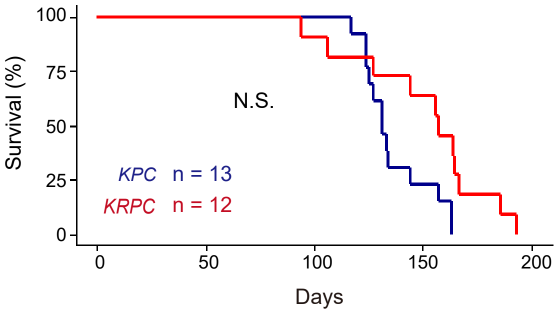

Supplemental Figure 3. Effects of Reck-deletion on survival of *KPC* mice. Kaplan-Meier survival curves for *KPC* and *KRPC* mice. N.S., log-rank test.

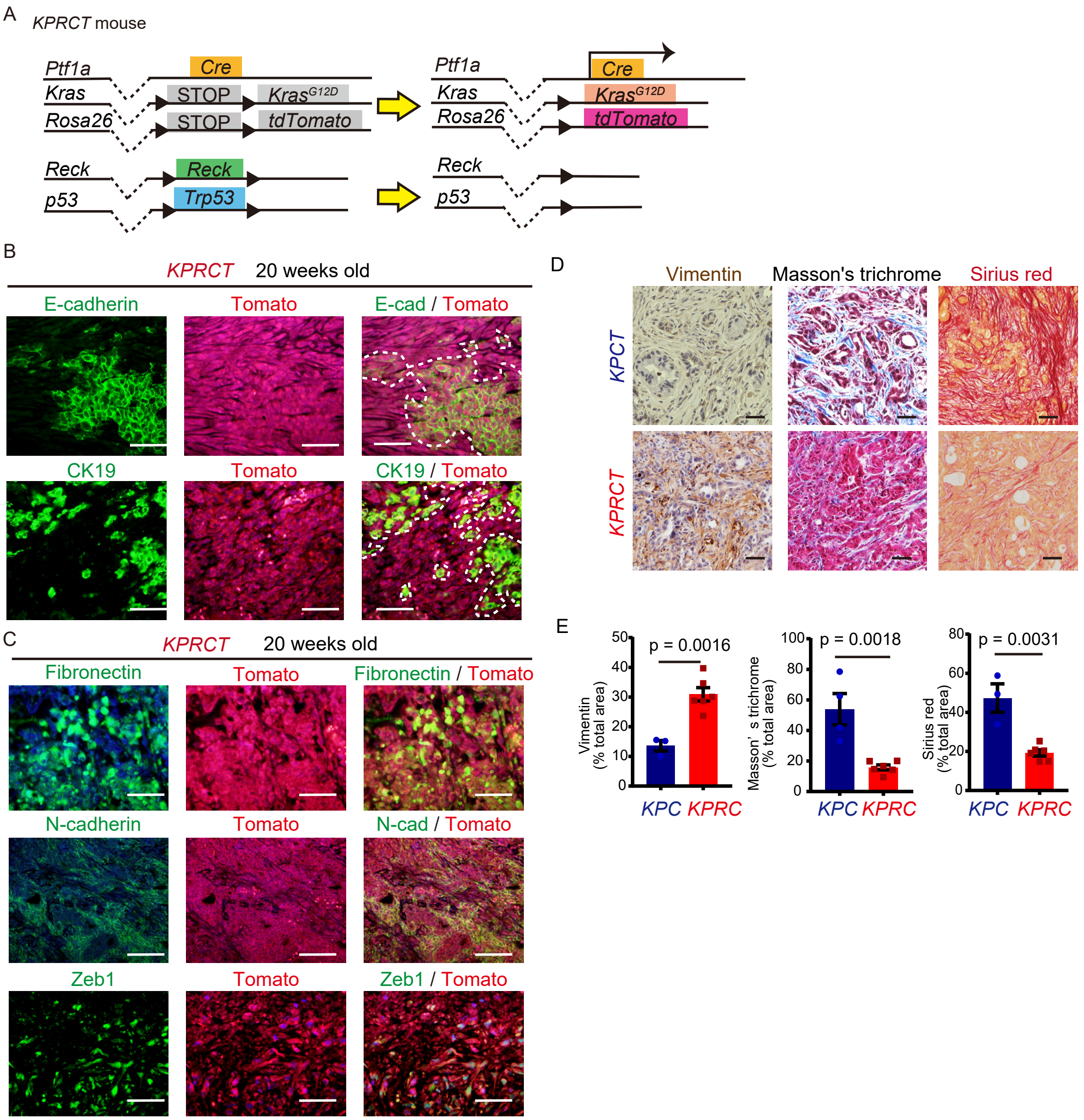

Supplemental Figure 4. Evidence that mesenchymal cells in Reck-null PDAC are originated from pancreatic epithelial cells in *KPRC* mice. (A) Schematic representation of Ptf1a-Cre-mediated recombination in pancreatic cells in the lineage tracing experiments using *Ptf1a-Cre; LSL-Kras^{G12D}; Reck$^{f/f}$; Trp53$^{f/+}$; LSL-Rosa$^{td\text{-}tomato}$ (KPRCT)*. Pancreatic epithelial cells are tagged by the red fluorescent protein tdTomato. (B) Immunofluorescent double staining for lineage marker Tomato (red; Ptf1a-expressing cells) and epithelial markers (green), E-cadherin or CK19, with nuclear counterstaining (blue) in PDAC sections from *KPRCT* mice. Images from the same field excited for green (epithelial cells) and red fluorescence (pancreatic epithelium-derived cells) are shown separately (left two panels) and overlaid (right panel) on images of blue fluorescence (nuclei). The white dotted line marks the border between an area of red cells and a cluster of yellow cells. (C) Immunofluorescent double staining for mesenchymal markers (green), fibronectin, N-cadherin, and Zeb1, with lineage marker Tomato (red) and nuclear counterstaining (blue) in PDAC sections from *KPRCT* mice. Red pancreatic epithelium-derived cells co-expressing green mesenchymal marker (fibronectin, N-cadherin, or Zeb1) are yellow in the overlay panels (right column), and such cells are abundant in PDAC developed in *KPRCT* mice. (D) Immunostaining for vimentin or stained with Masson' s trichrome or Sirius red. Upper panels: *Ptf1a-Cre; LSL-Kras^{G12D}; Trp53$^{f/+}$; LSL-Rosa$^{td\text{-}tomato}$ (KPCT)* mice. Lower panels: *KPRCT* mice. (E) Morphometric quantification of vimentin-positive area (pan-CAF), area stained blue with Masson' s trichrome (collagen fibers), and Sirius red-positive area (collagen fibers). Bar represents mean ± SEM of the data obtained from three sections each from three mice. Vimentin-positive area: p = 0.0016, Masson' s trichrome: p = 0.0018, Sirius red: p = 0.0031, Student' s t-test.

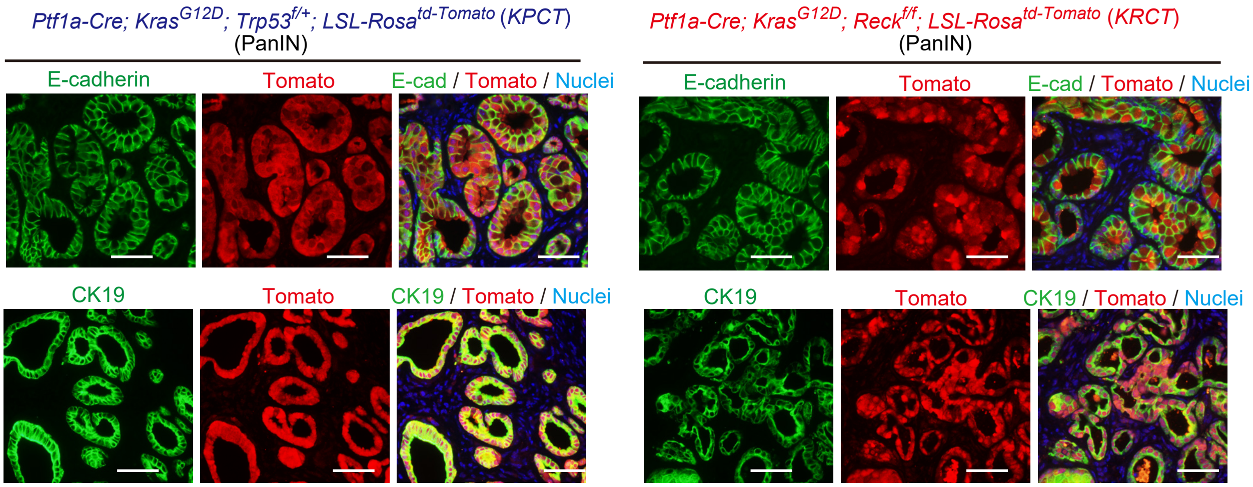

Supplemental Figure 5. Lineage tracing of the cells forming PanIN. No EMT occurred in the pancreatic epithelium-derived cells of PanIN in *KRCT* mice. Immunofluorescent double-staining of PanIN sections for lineage tracer (Tomato; red) and epithelial marker (green), E-cadherin or CK19 (scale: 50 µm). Left three columns: control *KPCT* mice. Right three columns: *KRCT* mice. Images of green fluorescence and red fluorescence are shown separately (left two panels) and overlaid with the image of nuclear counterstain (blue, rightmost panel of the three columns). Note that in overlaid images, red-positive/green-negative cells (indicating non-epithelial cells of pancreatic epithelial origin) are rare in PanIN in both groups of mice.

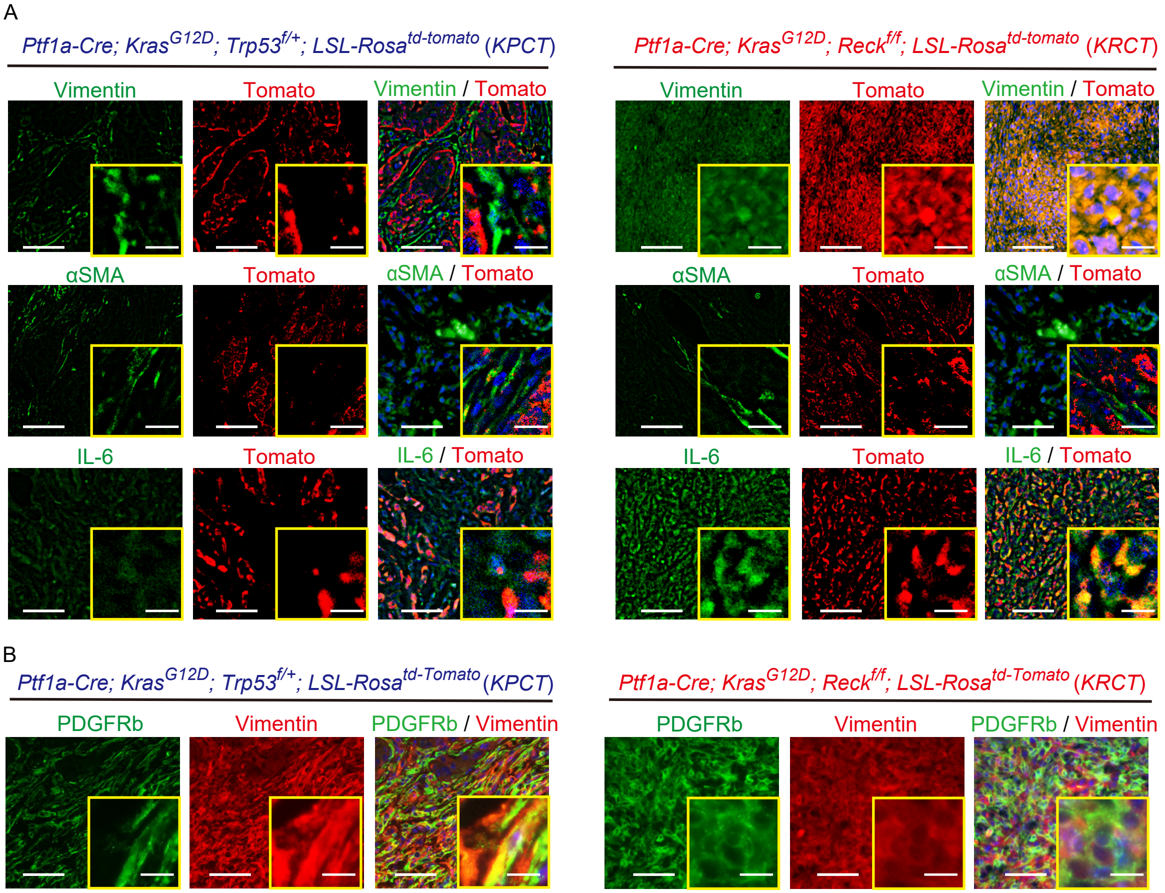

Supplemental Figure 6. Pancreatic Reck deletion gives rise to inflammatory CAF-like cells derived from pancreatic epithelial cells.(A) Detection of CAF markers in PDACs developed in the lineage tracer mice. Tissue sections as used in Figure 4B and Supplementary Figure 4B were double-stained for the lineage marker Tamato and a CAF markers (green), vimentin, α-SMA, or IL-6. Images are shown at two magnifications: low (scale: 50 µm) and high (inset, scale: 10 µm). Note that numerous red (pancreatic epithelium-derived) cells co-express the pan-CAF marker (vimentin, yellow signals) and iCAF marker (IL-6, yellow signals) but not myCAF marker (α-SMA) in PDAC developed in *KRCT* mice. (B) Immunofluorescent double-staining of PDAC sections for panCAF markers, vimentin (red) and PDGFRb (green). Left three columns: *KPCT* mice. Right three columns: *KRCT* mice. Images of green and red fluorescence are shown separately (left two panels of the respective three columns) and overlaid with nuclear counterstain (blue; right panel of the respective three columns; scale: 50 µm, inset, scale: 10 µm).

A

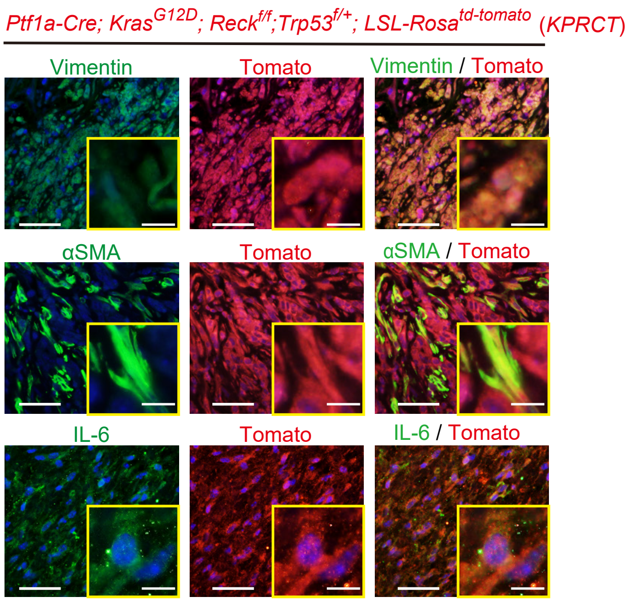

B

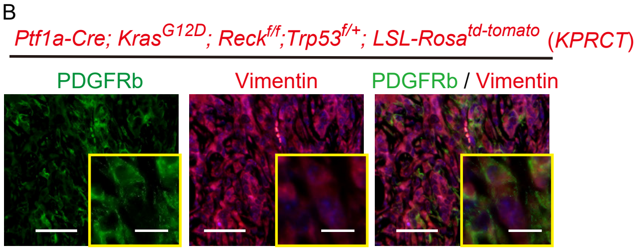

Supplemental Figure 7. Pancreatic Reck deletion gives rise to inflammatory CAF-like cells derived from pancreatic epithelial cells in KPC model. (A) Detection of CAF markers. Experiments similar to those shown in Figure 4B were performed using CAF markers (green): vimentin, α-SMA, and IL-6. Images are shown at two magnifications: low (scale: 50 μm) and high (inset, scale: 10 μm). Note that numerous red (pancreatic epithelium-derived) cells co-express the pan-CAF marker (vimentin, yellow signals) and iCAF markers (IL-6, yellow signals) but not myCAF marker (α-SMA) in PDAC developed in *KPRCT* mice. (B) Immunofluorescent double-staining of PDAC sections for vimentin (red) and PDGFRb (green). Images of green and red fluorescence are shown separately (left two panels of the respective three columns) and overlaid with nuclear counterstain (blue; right panel of the respective three columns). Images are shown at two magnifications: low (scale: 50 μm) and high (inset, scale: 10 μm).
